# Direct visualization of inner-sphere electrocatalytic reactions as they occur at detachable electrochemical interfaces

**DOI:** 10.1039/d6sc01821a

**Published:** 2026-04-27

**Authors:** Xiangbiao Zeng, Peimeng Qiu, Xi Cui, Chengbiao Zhu, Chen Zeng, Jianxiong Chen, Peng Li, Shengli Chen, Zhenwei Wei

**Affiliations:** a Hubei Key Lab. of Electrochemical Power Sources, College of Chemistry and Molecular Sciences, Wuhan University Wuhan 430072 China weizw2021@whu.edu.cn slchen@whu.edu.cn peng.li@whu.edu.cn

## Abstract

Molecular-level observation of inner-sphere electrocatalytic reactions has remained a longstanding challenge. Although recent advances in *in situ* and *operando* mass spectrometry enable probing of complex electrochemical processes, intrinsic limitations of mass spectrometry hinder characterization of adsorbed intermediates at inner-sphere interfaces. Herein, we report a detachable electrochemical interface strategy that enables direct visualization of inner-sphere electrocatalytic reactions as they occur. A positively charged [Au_9_(PPh_3_)_8_]^3+^ nanocluster acts as a molecular interfacial carrier that selectively assembles at the cathodic interface and mediates electron transfer. Under reaction potentials, changes in the charges of the cluster weaken its interaction with the electrode, allowing reaction information-bearing clusters to desorb into solution for immediate mass spectrometric analysis while retaining their interfacial origin. Based on this strategy, we achieved *operando* visualization of multiple types of inner-sphere electrocatalytic processes. In the hydrogen evolution reaction, we directly observed short-lived hydrogen intermediates on the [Au_9_(PPh_3_)_8_]^3+^ associated with proton-coupled electron transfer (PCET) pathways, with the interfacial species identified as [Au_9_H(PPh_3_)_8_]^2+^. In the reduction of nitrosobenzene, we captured the formation of nitrosobenzene radical anions at the interface ([Au_9_(PPh_3_)_7_ + PhNO]^2+^) and their evolution pathway in the solution phase. Additionally, in the interface reconstruction reaction, we observed the potential-dependent reconstruction and growth of [Au_9_(PPh_3_)_8_]^3+^ and [Au_11_(PPh_3_)_8_]^3+^ in the presence of metal ions, providing new insights into interfacial reconstruction phenomena. By converting previously elusive interfacial reactions into directly observable single chemical entities, this work establishes a general *operando* platform for probing short-lived inner-sphere electrocatalytic intermediates, bridging a critical gap between interfacial reaction chemistry and molecular-level detection.

## Abstract 2

A detachable electrochemical interface using [Au_9_(PPh_3_)_8_]^3+^ nanocluster enables real-time MS monitoring of inner-sphere electrocatalysis.

## Graphical abstract

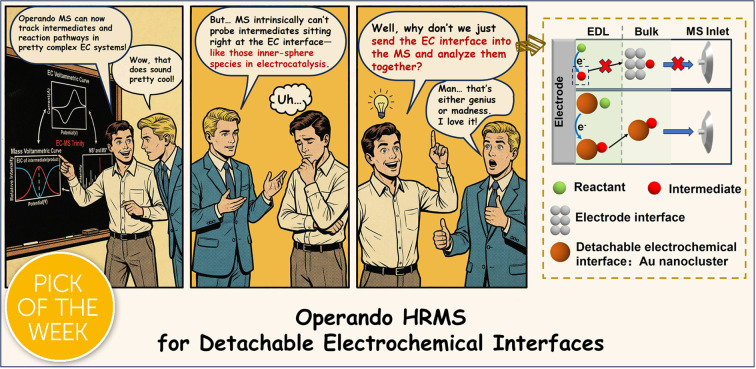

## Introduction

Electrocatalytic reactions play a central role in diverse electrochemical (EC) energy conversion and electrosynthesis processes, including the hydrogen evolution reaction,^1–4^ CO_2_ reduction,^5–8^ nitrate reduction,^9–12^ and organic electrosynthesis.^13–16^ In many of these reactions, the determining steps often follow an inner-sphere mechanism, where reactants or intermediates are required to adsorb onto the polarized electrode interface and complete electron and proton transfer in direct contact with the electrode.^11,17–19^ Unlike outer-sphere electron-transfer processes, inner-sphere electrocatalytic reactions inherently occur at the electrode–electrolyte interface, rendering key intermediates spatially restricted on the electrode interface and highly transient.^20,21^ Despite the extensive progress in electrocatalysis, most available characterization techniques focus either on the bulk solution or on electrode surface structures after the reaction, but cannot directly capture molecular-level information arising from interfacial reactions, making direct observation of inner-sphere processes at the molecular scale highly challenging.

In recent years, infrared spectroscopy,^22–24^ Raman spectroscopy,^25,26^ and various X-ray spectroscopic techniques^27–29^ have played important roles in probing electrochemical interfaces, enabling access to information on adsorbed species and their reaction trends. These spectroscopic techniques provide robust *in situ* characterization capabilities and have significantly advanced the understanding of electrochemical interfaces.^30–32^ However, the information provided by these techniques is often limited to functional-group signatures, and there remains a gap in their ability to accurately identify molecular structures. Additionally, they are susceptible to interference in complex reaction environments. Electrochemical measurements primarily depend on macroscopic current-potential responses to deduce interfacial reaction mechanisms and are well suited for providing kinetic information, yet remain limited in resolving molecular-scale dynamics evolution during inner-sphere interfacial reactions.^32–34^

Compared with these conventional characterization techniques, mass spectrometry (MS), with its high sensitivity and molecular specificity, has been increasingly applied in electrochemical research.^35,36^ Recently developed *in situ* EC-MS methods integrate the EC cell into an electrospray ionization (ESI) source, enabling real-time MS detection of EC products and intermediates.^37,38^ For example, MS strategies based on desorption electrospray ionization,^39,40^ induced ESI,^41,42^ bipolar ESI,^43,44^ floating electrochemical ESI,^45–47^ and voltammetry ESI^48–50^ have enabled substantial progress in mechanistic studies of outer-sphere reaction systems and electrochemical–thermochemical coupled reactions. However, for inner-sphere electrocatalytic processes, many key intermediates are stable only at the polarized electrode interface and rapidly transform or decay once removed from the interface.^6,17,51^ In this sense, MS faces a fundamental limitation: the species it can detect are inherently desorbed, whereas the chemical entities that govern reaction pathways are confined to the interface. This mismatch between what is detected and where the reaction actually occurs has long cast doubt on the applicability of MS to inner-sphere electrocatalytic studies.

Against this background, a key question arises: can the interface itself be treated as an analyzable entity and introduced into mass spectrometric detection, thereby enabling direct *operando* analysis of inner-sphere reaction processes? Recent advances in *in situ* secondary ion mass spectrometry (SIMS) offer important inspiration for this concept. By directly sampling the solid–liquid interface with a high-energy ion beam, SIMS has been employed to investigate various EC interfacial processes. For example, Zhu *et al.*^52^ captured the Au(OH) adsorption intermediate onto a gold electrode surface during ethanol oxidation and identified it as a key active site. Subsequently, they analyzed the solid-electrolyte interphase (SEI) of lithium-ion batteries under different charging potentials and proposed that its formation is closely related to the reduction of solvated Li^+^ in the inner layer of the electrical double layer.^53^ Long *et al.*^54^ also used SIMS to study organic reduction reactions at the electrode–electrolyte interface. However, the hard ionization process in SIMS can easily damage fragile adsorbed intermediates at the interface. As a result, its ability to resolve molecular details of inner-sphere reactions remains limited.

In this work, we propose and demonstrate a detachable interface electrochemical mass spectrometry (DI-EC-MS) strategy. We combine this strategy with our previously developed floating electrochemical electrospray mass spectrometry (FE-ESI-MS) platform^46^ for *operando* monitoring of inner-sphere electrocatalytic processes. We designed and synthesized a positively charged [Au_9_(PPh_3_)_8_]^3+^ nanocluster to mimic the outermost active atomic environment of an electrode interface. Under cathodic potentials, the [Au_9_(PPh_3_)_8_]^3+^ clusters selectively enrich and cover the cathode surface. Electron transfer at the interface is then directed through the clusters. As a result, inner-sphere reactions preferentially occur on the cluster surface rather than on the electrode itself. Following the interfacial reaction, the electronic state of the [Au_9_(PPh_3_)_8_]^3+^ cluster changes. Its interaction with the electrode becomes weaker. The reaction-information-bearing clusters can then detach into solution and be analyzed by mass spectrometry in real time. In this way, the reaction chemistry that is normally confined to the interface is effectively “sectioned” and converted into detectable molecular entities. This enables direct *operando* visualization of inner-sphere electrocatalytic reactions while preserving their interfacial origin.

Using the DI-EC-MS, we achieved *operando* probing of several types of inner-sphere electrocatalytic processes. These include the PCET process and organic reduction reactions involving interfacial adsorbed molecules. We directly observed hydrogen intermediates related to PCET on the [Au_9_(PPh_3_)_8_]^3+^ clusters. We also captured adsorbed intermediates formed by single-electron transfer of nitrosobenzene at the interface. At the same time, *in situ* EC-MS detected trace nitrosobenzene radical anions in the solution phase. This result reveals a full molecular pathway in which the species forms at the interface, partially detaches, and then evolves in solution. We further studied the potential-dependent restructuring and growth of [Au_9_(PPh_3_)_8_]^3+^ and [Au_11_(PPh_3_)_8_]^3+^ clusters in the presence of metal ions. These results provide new molecular-level evidence for the dynamic evolution of the electrode interface. By enabling *operando* visualization of short-lived inner-sphere intermediates, the detachable electrochemical interface provides a general framework to bridge interfacial reaction chemistry and molecular-level detection.

## Results and discussion

### Construction of a detachable electrochemical interface platform for *operando* mass spectrometric analysis

**Fig. 1:**
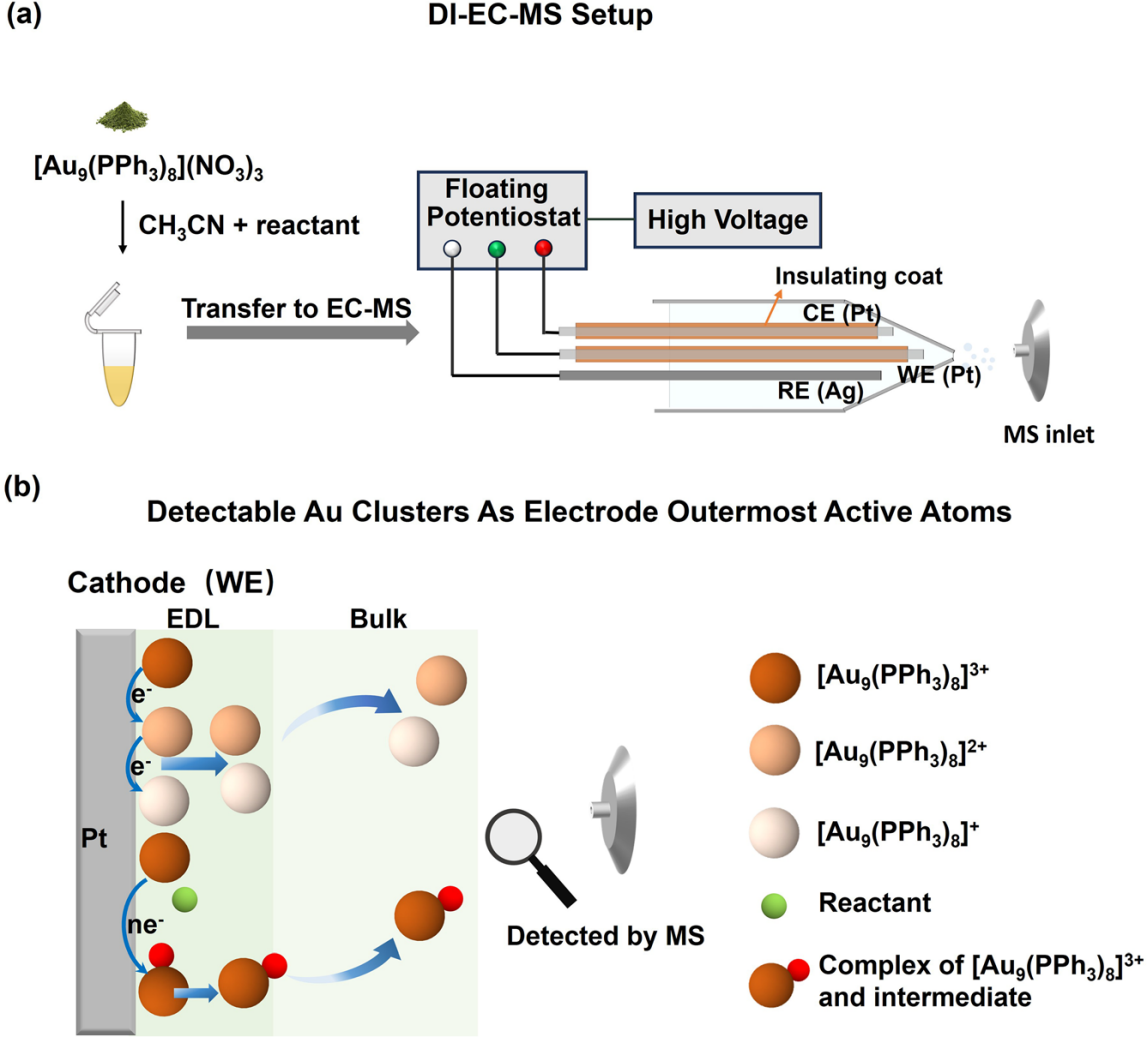
(a) Schematic illustration of the DI-EC-MS. In the experiment, [Au_9_(PPh_3_)_8_](NO_3_)_3_ and the target EC reaction substrates are dissolved in acetonitrile and then transferred into the online EC cell for continuous reaction monitoring. (b) Schematic illustration of detachable [Au_9_(PPh_3_)_8_]^3+^ clusters acting as the outermost active atomic layer of the working electrode. The negatively polarized Pt electrode electrostatically attracts the positively charged [Au_9_(PPh_3_)_8_]^3+^ clusters, leading to their enrichment on the electrode surface. After reduction of the clusters and the substrate molecules, the decreased positive charge weakens the interaction between the gold clusters and the interface, causing the clusters to desorb from the electrode surface and enter the solution.

As shown in Fig. 1a, the DI-EC-MS was adapted from our previously reported FE-ESI-MS device^46^ by introducing a floating potential control module, which allows the reaction potential to be adjusted with kilovolt-level high voltage. Therefore, the device can continuously monitor reactions during potential scanning (see SI Experimental Section 1.3 and Fig. S1 for details). We then placed the pre-synthesized gold nanocluster [Au_9_(PPh_3_)_8_]^3+^ together with EC reactants into the EC cell of this device. During cathodic polarization (Fig. 1b), [Au_9_(PPh_3_)_8_]^3+^ enters the electric double layer (EDL) and adsorbs tightly onto the electrode surface through electrostatic interaction. Because the electrode surface is largely covered by [Au_9_(PPh_3_)_8_]^3+^, the reactant molecules preferentially adsorb onto the clusters rather than directly onto the electrode. Electron transfer then occurs between the electrode and the reactants through the [Au_9_(PPh_3_)_8_]^3+^ clusters. After the [Au_9_(PPh_3_)_8_]^3+^ clusters and the adsorbed molecules receive electrons from the electrode, the positive charge of the clusters decreases and their interaction with the electrode becomes weaker. As a result, the clusters (“[Au_9_(PPh_3_)_8_]^3+^ reaction interface”) detach from the electrode along with the corresponding inner-sphere EC reaction intermediate information, and are directly sent to the MS for *operando* analysis.

### Synthesis and characterization of detachable interface materials

**Fig. 2:**
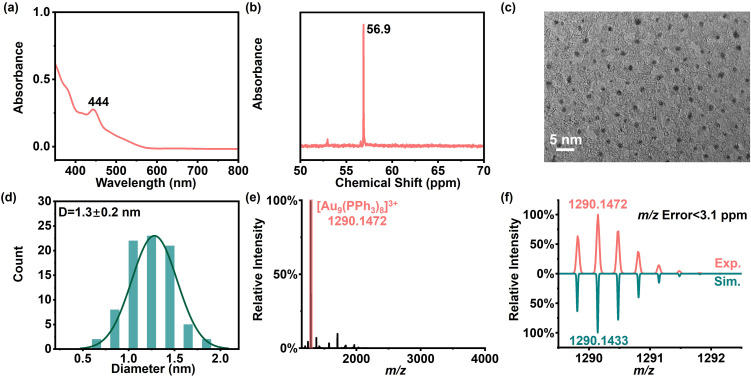
Characterization of [Au_9_(PPh_3_)_8_](NO_3_)_3_: (a) UV-vis spectrum; (b) ^31^P NMR spectrum; (c) TEM image; (d) particle size distribution histogram; (e) nESI-MS spectrum; (f) simulated and experimental isotope distribution patterns of [Au_9_(PPh_3_)_8_].

We compared gold nanoparticles (Au NPs) and the gold nanocluster [Au_9_(PPh_3_)_8_](NO_3_)_3_ as model materials for the EC reaction interface. Both Au NPs and [Au_9_(PPh_3_)_8_](NO_3_)_3_ were synthesized following reported procedures (SI, Experimental Section 1.5).^55,56^ The dispersion of Au NPs in water shows a wine-red color. Its UV-vis spectrum displays a characteristic absorption peak at 520 nm (Fig. S2a), confirming successful synthesis. However, in nanoelectrospray mass spectrometry (nESI-MS), no characteristic Au NP signals were observed in either positive ion mode (Fig. S2b) or negative ion mode (Fig. S2c). Transmission electron microscopy (TEM) image (Fig. S2d) and the particle size distribution (Fig. S2e) show that the Au NPs are relatively large (see Supplementary Note in the SI). Their large size and mass likely exceed the mass detection range of high resolution MS (HRMS). In contrast, the [Au_9_(PPh_3_)_8_](NO_3_)_3_ nanocluster shows a characteristic UV-vis absorption peak near 444 nm (Fig. 2a). Its ^31^P NMR spectrum shows a strong signal at 56.9 ppm (Fig. 2b). These results agree well with previous reports.^56,57^ TEM image (Fig. 2c) and particlesize analysis (Fig. 2d) indicate an average cluster size of about 1 nm, which is much smaller than that of the Au NPs. In HRMS, a base peak at *m*/*z* 1290.1472 is observed (Fig. 2e). Based on the exact mass and isotope pattern match (Fig. 2f), this peak is assigned to [Au_9_(PPh_3_)_8_]^3+^ (theoretical *m*/*z* 1290.1433; error < 3.1 ppm). This result confirms that the [Au_9_(PPh_3_)_8_]^3+^ cluster can be accurately measured by HRMS. Therefore, compared with Au NPs, [Au_9_(PPh_3_)_8_](NO_3_)_3_ is more suitable as a model material for the EC reaction interface.

### Feasibility evaluation of the DI-EC-MS strategy

**Fig. 3:**
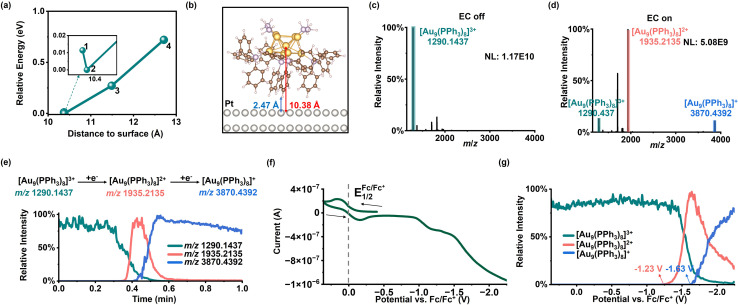
(a) Relative energy profile of [Au_9_(PPh_3_)_8_]^3+^ at different distances from the Pt electrode surface; the inset shows a locally enlarged view. (b) Schematic diagram of the relative configuration of [Au_9_(PPh_3_)_8_]^3+^ and the Pt surface at position 2. (c) MS of [Au_9_(PPh_3_)_8_]^3+^ before electrolysis. (d) MS of [Au_9_(PPh_3_)_8_]^3+^ after electrolysis. (e) EICs of three charge states of the gold cluster during electrolysis. (f) Online CV of [Au_9_(PPh_3_)_8_]^3+^ solution obtained using the FE-ESI-MS. (g) MSV curves of selected ions during the EC scan. Experimental conditions: acetonitrile solvent; [Au_9_(PPh_3_)_8_](NO_3_)_3_ (500 ppm); Fc (0.1 mM); scan rate: 20 mV s^−1^.

To demonstrate that [Au_9_(PPh_3_)_8_]^3+^ can tightly adsorb onto the electrode surface and act as the outermost active atomic layer during EC reactions, we first computationally simulated the distance of the [Au_9_(PPh_3_)_8_]^3+^ cluster from the Pt electrode surface. To reduce the computational cost while maintaining accuracy in describing interfacial interactions, we employed an asymmetric ligand model for [Au_9_(PPh_3_)_8_]^3+^: the complete triphenylphosphine (PPh_3_) ligands were retained on the side facing the Pt surface, whereas PH_3_ was used as an approximation on the distal side. Using this model, we performed geometry optimization for configurations of [Au_9_(PPh_3_)_8_]^3+^ positioned at various normal distances (*z*) above the Pt electrode surface (see SI, Experimental Section 1.6 for details). Specifically, initial distances between the gold core and the Pt surface were set to 9.5, 11.0, 12.0, and 13.0 Å, and each configuration was structurally optimized to obtain its corresponding local minimum energy geometry. As shown in Fig. 3a, when the initial distance was 11.0 Å, optimization converged to 10.38 Å (position 2), and this configuration exhibited the lowest energy. Notably, when the initial distance was closer (9.5 Å), the system also returned to a position near 10.38 Å during optimization (position 1, nearly overlapping with position 2); the structural evolution process is depicted in Fig. S3. In contrast, when the initial heights were 11.0 and 12.0 Å, optimization converged to 11.49 Å and 12.71 Å, respectively (positions 3 and 4), with energies significantly higher than that of position 2. Thus, the distance of 10.38 Å from the electrode surface represents the lowest-energy configuration for [Au_9_(PPh_3_)_8_]^3+^ on the Pt electrode surface. At this configuration, the hydrogen atoms of the PPh_3_ ligands on the [Au_9_(PPh_3_)_8_]^3+^ cluster were only 2.47 Å from the electrode surface (Fig. 3b), indicating that this cationic cluster can enter the inner layer of EDL and establish an intimate contact with the electrode surface. These results indicate that [Au_9_(PPh_3_)_8_]^3+^ interacts strongly with the Pt surface and is primarily physisorbed.

We next investigated whether this interface exhibits electron transfer properties. We first examined the reduction behavior of [Au_9_(PPh_3_)_8_]^3+^ under a constant voltage of −2.5 V (with two platinum wires as the working electrode and counter electrode and Ag/AgCl as the reference electrode). HRMS spectra acquired before and after EC reactions (Fig. 3c and d) were compared. Prior to the reaction, the MS spectrum displayed only the signal corresponding to [Au_9_(PPh_3_)_8_]^3+^ (*m*/*z* 1290.1437). After electrolysis, the intensity of the [Au_9_(PPh_3_)_8_]^3+^ signal decreased markedly, while signals attributable to [Au_9_(PPh_3_)_8_]^2+^ (*m*/*z* 1935.2135; theoretical *m*/*z* 1935.2153) and [Au_9_(PPh_3_)_8_]^+^ (*m*/*z* 3870.4392; theoretical *m*/*z* 3870.4311) increased substantially (see Fig. S4a and b for isotopic pattern matching). These results demonstrate that the [Au_9_(PPh_3_)_8_]^3+^ clusters can undergo single- and multi-electron transfer processes with the electrode. After accepting electrons, the [Au_9_(PPh_3_)_8_]^3+^ clusters, serving as a detachable “electrode interface”, can directly enter the solution and be detected by mass spectrometry. We further compared HRMS spectra obtained after EC reactions in different solvent systems. The results indicated that for the aprotic solvents, including acetonitrile, acetone, dimethyl sulfoxide, and *N*,*N*-dimethylformamide, reduction signals corresponding to electron transfer of the gold clusters were observable in the MS (Fig. 3d and S5a–c), with acetonitrile giving the best response. In contrast, protic solvents (Fig. S6a–c), including methanol, ethanol, and acetonitrile–water (9 : 1, v/v), failed to produce reduction signals of the gold clusters. This is likely due to competitive surface adsorption from the hydrogen evolution reaction. Accordingly, acetonitrile was employed as the solvent in all subsequent experiments.

We further selected the *m*/*z* values of [Au_9_(PPh_3_)_8_]^3+^, [Au_9_(PPh_3_)_8_]^2+^, and [Au_9_(PPh_3_)_8_]^+^ and plotted the extracted ion chronograms (EICs) as a function of time (Fig. 3e). The trends are clearer in this plot: as the reduction proceeds, the signal of [Au_9_(PPh_3_)_8_]^3+^ gradually decreases, while the signals of [Au_9_(PPh_3_)_8_]^2+^ first increased and then decreased over time. Finally, the signal of [Au_9_(PPh_3_)_8_]^+^ began to increase. To obtain more reliable and comparable potential information, we added ferrocene as an internal standard and performed cyclic voltammetry (CV) scanning using a three-electrode FE-ESI-MS device (Fig. 1a). The electrode potential scale was calibrated using the half-wave potential of the ferrocene redox couple (Fc/Fc^+^) measured by online CV scanning (Fig. 3f).^50,58^ The potential-dependent behaviors of [Au_9_(PPh_3_)_8_]^3+^, [Au_9_(PPh_3_)_8_]^2+^, and [Au_9_(PPh_3_)_8_]^+^ were then plotted as extracted ion intensity *versus* potential (Fig. 3g), *i.e.*, mass spectrometric voltammograms (MSV, by analogy with CV in traditional EC research). From the MSV curves, when the reduction potential of [Au_9_(PPh_3_)_8_]^2+^ is reached (−1.23 V *vs.* Fc/Fc^+^), the [Au_9_(PPh_3_)_8_]^2+^ signal increases, while the [Au_9_(PPh_3_)_8_]^3+^ signal decreases. At more negative potential, near the reduction potential of [Au_9_(PPh_3_)_8_]^+^ (−1.63 V *vs.* Fc/Fc^+^), the [Au_9_(PPh_3_)_8_]^+^ signal increases and the [Au_9_(PPh_3_)_8_]^2+^ signal correspondingly decreases. These results show that the single-electron and multi-electron transfer processes at the interface are strongly potential-dependent. Collectively, these data confirm that we have established a detachable electrochemical interface that enables real-time monitoring of interfacial electron-transfer processes by *in situ* EC-MS.

### Investigation of typical inner-sphere electrocatalysis using DI-EC-MS

To demonstrate that DI-EC-MS can serve as a powerful molecular-level tool for inner-sphere electrocatalytic chemistry research, we systematically examined three representative interfacial scenarios: (i) transient interfacial hydrogen intermediates in the PCET process, (ii) potential-dependent restructuring of [Au_9_(PPh_3_)_8_]^3+^ and [Au_11_(PPh_3_)_8_]^3+^ clusters under cathodic polarization and its interfacial-to-solution evolution, and (iii) single-electron inner-sphere reduction of nitrosobenzene. Together, these studies establish that the DI-EC-MS not only captures short-lived reaction intermediates, but also resolves dynamic reaction pathways and interfacial structural evolution in an *operando* manner.

### Direct molecular identification of interface-confined hydrogen intermediates in inner-sphere PCET

**Fig. 4:**
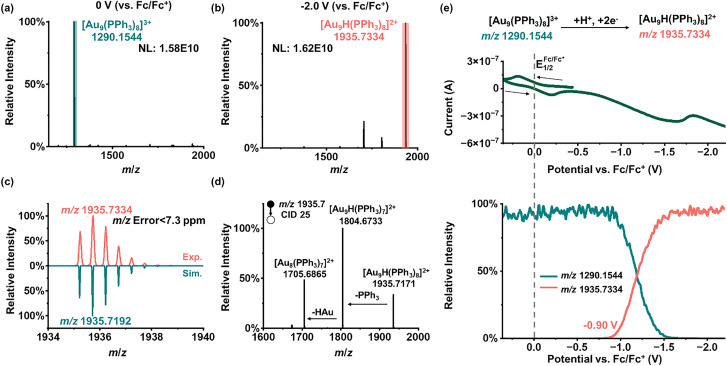
EC reaction of a mixed solution of [Au_9_(PPh_3_)_8_](NO_3_)_3_ and HCOOH was monitored using the DI-EC-MS. (a) MS of the mixed solution at 0 V *vs.* Fc/Fc^+^. (b) MS at −2.0 V *vs.* Fc/Fc^+^. (c) Experimental and simulated isotope distribution patterns of [Au_9_H(PPh_3_)_8_]^2+^. (d) Tandem MS of [Au_9_H(PPh_3_)_8_]^2+^. (e) Online CV and corresponding MSV curves. Experimental conditions: acetonitrile solvent; [Au_9_(PPh_3_)_8_](NO_3_)_3_ (500 ppm); HCOOH (1 mM); Fc (0.1 mM); scan rate: 20 mV s^−1^.

Hydrogen-involving intermediates are central to proton-coupled electron transfer at electrified interfaces, but interfacial changes during this process are difficult to capture directly, and most studies instead can only detect H_2_.^59,60^ We used formic acid as a proton source and attempted to observe this interfacial process on the [Au_9_(PPh_3_)_8_]^3+^ cluster. As shown in Fig. 4a, the HRMS spectrum at 0 V (*vs.* Fc/Fc^+^) is still dominated by the base peak of [Au_9_(PPh_3_)_8_]^3+^. After electrolysis, a new intense peak appears at *m*/*z* 1935.7334 (Fig. 4b). Based on the accurate mass and isotope pattern (Fig. 4c), this peak is assigned to a H adsorption intermediate on the Au cluster, [Au_9_H(PPh_3_)_8_]^2+^ (theoretical *m*/*z* 1935.7192). From the online CV and MSV plots (Fig. 4e), it can be observed that the signal intensity of this species increases rapidly near −0.90 V (*vs.* Fc/Fc^+^). The initial cluster carries three positive charges, while H^+^ has one positive charge, and the product [Au_9_H(PPh_3_)_8_]^2+^ carries two positive charges, indicating that two electrons are gained from the electrode during this process. As the hydrogen at this stage is in a reduced state and PPh_3_ is unlikely to bind such reduced hydrogen, we infer that hydrogen binds to gold rather than to PPh_3_. This result is also further supported by the loss of AuH in the tandem MS spectrum of [Au_9_H(PPh_3_)_8_]^2+^ (Fig. 4d) and agrees with the prior reports.^57,61^ We further confirmed this assignment using deuterated formic acid (DCOOD). The CV and MSV curves during electrolysis (Fig. S7a) are very similar to those with HCOOH. The MS spectra at 0 V and −2.0 V (*vs.* Fc/Fc^+^) (Fig. S7b and c) show a new peak at *m*/*z* 1936.2365, assigned to [Au_9_D(PPh_3_)_8_]^2+^ (theoretical *m*/*z* 1936.2223), which is 0.5 Th (1 Da) higher than that of [Au_9_H(PPh_3_)_8_]^2+^. Its experimental isotope distribution pattern (Fig. S7d) matches the simulated distribution, and the tandem MS spectrum (Fig. S7e) shows the corresponding neutral loss of AuD.

We then compared this reaction with different concentrations of formic acid. When the concentration of HCOOH is 0, no signal of [Au_9_H(PPh_3_)_8_]^2+^ is observed; when the concentration of formic acid (HCOOH) is 1–10 µM, only noise-level signals are detected, and these signals subsequently become unobservable with the appearance of [Au_9_(PPh_3_)_8_]^2+^. As shown in Fig. S8a, when the concentration increases from 100 µM to 100 mM, the [Au_9_H(PPh_3_)_8_]^2+^ signal increases rapidly near −0.90 V (*vs.* Fc/Fc^+^). Although the appearance potential is similar across these concentrations, the signal intensity differs (Fig. S8b). As the HCOOH concentration increases, the intensity of [Au_9_H(PPh_3_)_8_]^2+^ first increases and then decreases, reaching a maximum at 1 mM. At higher acid concentrations, the signal drops, likely because excess protons compete with [Au_9_(PPh_3_)_8_]^3+^ for adsorption onto the Pt cathode, which prevents the cluster from approaching the electrode surface efficiently. We also monitored the behaviors of [Au_9_(PPh_3_)_8_]^2+^ and [Au_9_(PPh_3_)_8_]^+^ under these conditions. As shown in Fig. S9a, with 0–10 µM HCOOH, [Au_9_(PPh_3_)_8_]^2+^ appears at about −1.25 V, with 100 µM HCOOH, it shifts to about −1.40 V, and with 1–100 mM HCOOH, no [Au_9_(PPh_3_)_8_]^2+^ signal is detected. A similar trend is observed for [Au_9_(PPh_3_)_8_]^+^ (Fig. S9b): the signal appears near −1.65 V with 0–100 µM HCOOH but disappears at 1–100 mM concentration. These results indicate that when Pt is used as the cathode, excessive proton concentration competes with [Au_9_(PPh_3_)_8_]^3+^ for surface adsorption and can even fully block electron transfer between the cluster and the electrode. The MSV curves of different charge state clusters further show that [Au_9_H(PPh_3_)_8_]^2+^ is formed at a more positive potential than [Au_9_(PPh_3_)_8_]^2+^ and [Au_9_(PPh_3_)_8_]^+^. This excludes the possibility that [Au_9_H(PPh_3_)_8_]^2+^ originates from a recombination of [Au_9_(PPh_3_)_8_]^2+^ with H˙ generated at the Pt electrode, or from association of [Au_9_(PPh_3_)_8_]^+^ with H^+^. Instead, [Au_9_H(PPh_3_)_8_]^2+^ is more likely an H^−^ adsorbed intermediate formed *via* two-electron transfer at the [Au_9_(PPh_3_)_8_]^3+^ interface. Furthermore, in previously reported gold clusters containing hydrogen, the hydrogen is also regarded as H^−^.^57^ This suggests that the interfacial H* is more consistent with hydride (H^−^) rather than the hydrogen radical (H˙). On Pt electrodes, the hydrogen evolution reaction is commonly described by adsorbed H˙ intermediates,^19,62^ whereas Au generally requires a more negative overpotential than Pt,^63^ which makes H^−^ type adsorption more likely. Moreover, the active sites on the Pt surface may be occupied by adsorbed [Au_9_(PPh_3_)_8_]^3+^ clusters, thereby reducing the likelihood of H^+^ accessing exposed Pt active sites. These results indicate that the observed H^−^ species is more likely generated on the Au clusters.

### Electrode interfacial reconstruction under cathodic polarization: Au cluster growth

Electrode interface reconstruction is also a fundamental issue in HER studies and is closely related to maintaining high catalytic efficiency.^64^ Because the synthesized [Au_9_(PPh_3_)_8_](NO_3_)_3_ solution contains Au(PPh_3_)^+^ precursor ions, we further examined how metal ions affect electrode interfacial reconstruction during the HER. During the reduction of a solution containing 1 mM HCOOH and 500 ppm [Au_9_(PPh_3_)_8_](NO_3_)_3_, in addition to the [Au_9_H(PPh_3_)_8_]^2+^ signal, a new peak at *m*/*z* 2033.7155 is observed in the HRMS (Fig. S10a and b). Based on the accurate mass and isotope distribution pattern (Fig. S10c), this peak is assigned to a cluster-growth intermediate, [Au_10_(PPh_3_)_8_]^2+^ (theoretical *m*/*z* 2033.6985). The MSV plot (Fig. S10d) shows that this species appears nearly at −0.5 V (*vs.* Fc/Fc^+^). We think it may be transformed from [Au_9_(PPh_3_)_8_]^3+^. When the HCOOH concentration is increased to 10 mM, the intensity of [Au_10_(PPh_3_)_8_]^2+^ increases (Fig. S10e). By comparing the MSV plots of [Au_10_(PPh_3_)_8_]^2+^ at HCOOH concentrations of 1 mM and 10 mM, it can be observed that [Au_10_(PPh_3_)_8_]^2+^ at a HCOOH concentration of 1 mM exhibits a trend of first increasing and then decreasing, whereas at a HCOOH concentration of 10 mM, it shows a trend of continuous enhancement (Fig. S10d and f). This indicates that a concentration of 10 mM HCOOH may be more suitable for the formation of this cluster.

**Fig. 5:**
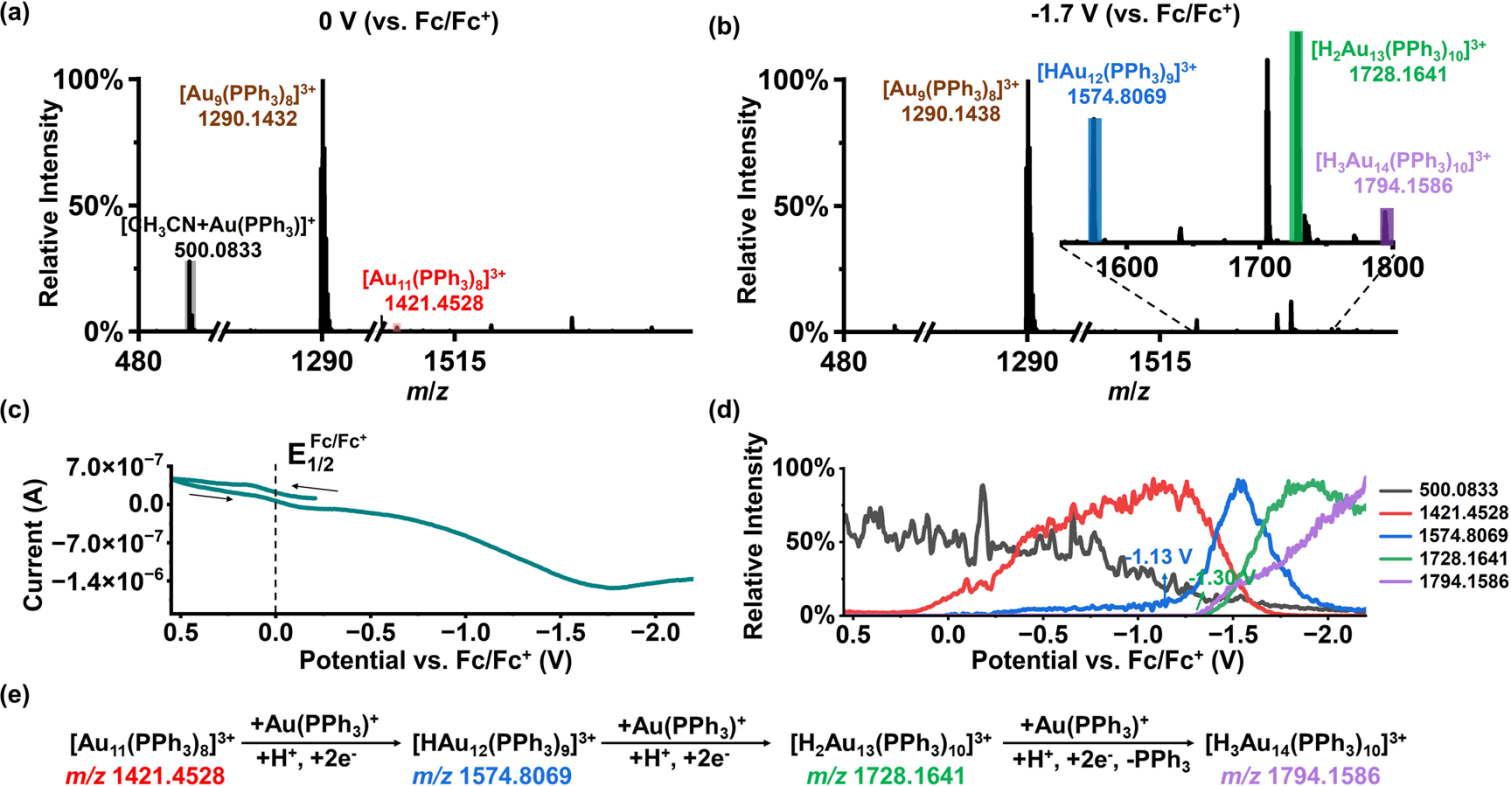
EC reaction of a mixed solution of [Au_9_(PPh_3_)_8_](NO_3_)_3_, HCOOH, and Au(PPh_3_)NO_3_ monitored using DI-EC-MS. (a) MS at 0 V *vs.* Fc/Fc^+^. (b) MS at −1.7 V *vs.* Fc/Fc^+^. (c) Online CV during the EC scan. (d) MSV curve during the EC scan. (e) Proposed growth mechanism from [Au_11_(PPh_3_)_8_]^3+^ to [H_3_Au_14_(PPh_3_)_10_]^3+^. Experimental conditions: acetonitrile solvent; [Au_9_(PPh_3_)_8_](NO_3_)_3_ (500 ppm); HCOOH (10 mM); Au(PPh_3_)NO_3_ (0.5 mM); Fc (0.1 mM); scan rate: 20 mV s^−1^.

We consider the observed cluster growth to be closely related to reconstruction at the electrode interface. To further test this idea, we added 0.5 mM Au(PPh_3_)NO_3_ to a solution containing 10 mM HCOOH and 500 ppm [Au_9_(PPh_3_)_8_](NO_3_)_3_. Surprisingly, even before applying any electrochemical potential, a signal at *m*/*z* 1421.4525 is already observed (Fig. S11a and b). Based on the accurate mass and isotope distribution pattern (Fig. S11c), this peak is assigned to [Au_11_(PPh_3_)_8_]^3+^. This species likely forms because Au(PPh_3_)^+^ disproportionates under acidic conditions to generate Au(0), which then combines with [Au_9_(PPh_3_)_8_]^3+^. Previous studies have reported that Au(PPh_3_)NO_3_ can readily decompose to elemental Au.^65,66^ As shown in Fig. S11d, the intensity of [Au_11_(PPh_3_)_8_]^3+^ increases with increasing formic acid concentration, indicating that a higher acid concentration promotes Au(PPh_3_)NO_3_ decomposition and cluster growth. When this solution is further applied to EC reactions (Fig. 5a and b), three new peaks appear after electrolysis. From the accurate masses and isotope distribution patterns (Fig. S12a–c), they are assigned to [HAu_12_(PPh_3_)_9_]^3+^ (*m*/*z* 1574.8069; theoretical *m*/*z* 1574.8095), [H_2_Au_13_(PPh_3_)_10_]^3+^ (*m*/*z* 1728.1641; theoretical *m*/*z* 1728.1647), and [H_3_Au_14_(PPh_3_)_10_]^3+^ (*m*/*z* 1794.1586; theoretical *m*/*z* 1794.1562). The CV and MSV plots (Fig. 5c and d) show that the signal intensity of [HAu_12_(PPh_3_)_9_]^3+^ increases rapidly near −1.13 V and then decreases, while [H_2_Au_13_(PPh_3_)_10_]^3+^ and [H_3_Au_14_(PPh_3_)_10_]^3+^ appear near −1.30 V. The values 500.0833 and 1421.4528 in the MSV plot correspond to the substrates [CH_3_CN + Au(PPh_3_)]^+^ and [Au_11_(PPh_3_)_8_]^3+^, respectively. The structure of [CH_3_CN + Au(PPh_3_)]^+^ was determined by high-resolution mass spectrometry based on its exact mass-to-charge ratio and isotopic distribution pattern, as shown in Fig. S12d. By comparing the molecular formulae of clusters of varying sizes, we find that the clusters grow stepwise by addition of one HAu unit each time (Fig. 5e). Specifically, [Au_11_(PPh_3_)_8_]^3+^ undergoes progressive transformation *via* Au–H-mediated reduction to [HAu_12_(PPh_3_)_9_]^3+^, [H_2_Au_13_(PPh_3_)_10_]^3+^, and [H_3_Au_14_(PPh_3_)_10_]^3+^, confirming the crucial role of Au–H in cluster growth and electrode interface reconstruction. To verify that these species indeed contain the corresponding number of H^−^, deuterium labeling experiments were performed using DCOOD in place of HCOOH. Mass spectra acquired at 0 V and −1.7 V (*vs.* Fc/Fc^+^), together with isotopic distribution patterns (Fig. S13a–e), demonstrated that [HAu_12_(PPh_3_)_9_]^3+^, [H_2_Au_13_(PPh_3_)_10_]^3+^, and [H_3_Au_14_(PPh_3_)_10_]^3+^ were converted to [DAu_12_(PPh_3_)_9_]^3+^, [D_2_Au_13_(PPh_3_)_10_]^3+^, and [D_3_Au_14_(PPh_3_)_10_]^3+^, respectively, with corresponding shifts in *m*/*z* values. The CV and MSV plots (Fig. S13f and g) showed trends similar to those obtained with HCOOH, and the clusters grew stepwise by addition of one DAu unit (Fig. S13h).

### Mapping the interface-to-solution evolution of the nitrosobenzene radical anion

**Fig. 6:**
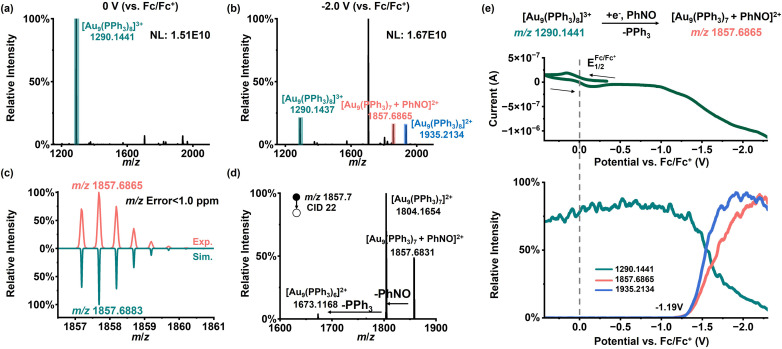
EC reaction of a mixed solution of [Au_9_(PPh_3_)_8_](NO_3_)_3_ and PhNO was monitored using the DI-EC-MS. (a) MS at 0 V *vs.* Fc/Fc^+^. (b) MS at −2.0 V *vs.* Fc/Fc^+^. (c) Experimental and simulated isotope distribution patterns of [Au_9_(PPh_3_)_7_ + PhNO]^2+^. (d) Tandem MS of [Au_9_(PPh_3_)_7_ + PhNO]^2+^. (e) CV and corresponding MSV curves during the EC scan. Experimental conditions: acetonitrile solvent; [Au_9_(PPh_3_)_8_](NO_3_)_3_ (500 ppm); PhNO (1 mM); Fc (0.1 mM); scan rate: 20 mV s^−1^.

While hydrogen intermediates represent archetypal proton-coupled electron transfer processes, inner-sphere single-electron reductions raise an additional mechanistic question: how a radical intermediate generated at the interface partially escape into solution? We carried out EC reactions using nitrosobenzene (PhNO) together with [Au_9_(PPh_3_)_8_](NO_3_)_3_. As shown in Fig. 6a and b, after electrolysis, a new peak appears at *m*/*z* 1857.6865, which is assigned to [Au_9_(PPh_3_)_7_ + PhNO]^2+^. The isotope distribution pattern (Fig. 6c) and tandem MS data (Fig. 6d) support this assignment. Fragmentation of *m*/*z* 1857.7 produces an ion at *m*/*z* 1804.1654, assigned to [Au_9_(PPh_3_)_7_]^2+^ (theoretical *m*/*z* 1804.1697), corresponding to the neutral loss of a PhNO fragment. As shown in the CV and MSV plots (Fig. 6e), [Au_9_(PPh_3_)_7_ + PhNO]^2+^ appears at nearly the same potential as [Au_9_(PPh_3_)_8_]^2+^, around −1.19 V (*vs.* Fc/Fc^+^). The agreement in reduction potentials suggests that this single-electron transfer process likely proceeds in a stepwise manner at the electrode interface. Under deep interfacial polarization, electron transfer first occurs from the Pt electrode to [Au_9_(PPh_3_)_8_]^3+^, generating [Au_9_(PPh_3_)_8_]^2+^. The electron is then further transferred through adsorbed PhNO on [Au_9_(PPh_3_)_8_]^2+^, accompanied by loss of one PPh_3_ ligand. We therefore propose that adsorbed nitrosobenzene at the interface tends to form the radical anion PhNO˙^−^. Because PhNO is a common substrate in EC coupling reactions of nitroaromatics,^67,68^ we further searched for PhNO˙^−^ using negative ion mode FE-ESI-MS. As shown in Fig. S14a, a signal at *m*/*z* 107.0377 appears after electrolysis, which matches well with the theoretical *m*/*z* 107.0376 of PhNO˙^−^. The MSV plot (Fig. S14b) shows that this species forms at a more negative potential than [Au_9_(PPh_3_)_7_ + PhNO]^2+^, indicating that it likely originates from species that detach from the cluster-based interface and then enter the solution. This finding challenges the common view that EC coupling between nitroaromatics and arylboronic acids proceeds mainly through outer-sphere pathways.^46,69,70^ Based on our previous *in situ* EC mass spectrometry studies, further reduction products of PhNO can include nitrene ([M + H]^+^, theoretical *m*/*z* 92.0495), aniline (M˙^+^, theoretical *m*/*z* 93.0573) and phenylhydroxylamine ([M + H]^+^, theoretical *m*/*z* 110.0601).^46,69^ However, these products are not observed under the present conditions (Fig. S15a and b), likely because the solution lacks sufficient proton sources for hydrogenation steps. Accordingly, upon addition of 1 mM HCOOH to the system, nitrene ([M + H]^+^, *m*/*z* 92.0501), aniline (M˙^+^, *m*/*z* 93.0579), and phenylhydroxylamine ([M + H]^+^, *m*/*z* 110.0609) began to appear at approximately −0.5 V (Fig. S16a–d). At this formic acid concentration, [Au_9_(PPh_3_)_8_]^2+^ was not generated (Fig. S9a), and consequently, [Au_9_(PPh_3_)_7_ + PhNO]^2+^ was not observed (Fig. S17). This result indicates that formation of PhNO˙^−^ is favored under aprotic conditions, which provides useful guidance for reactions that proceed through a PhNO˙^−^ mechanism.

Although the current work primarily relies on the [Au_9_(PPh_3_)_8_]^3+^ nanocluster as the interfacial carrier, we believe that this system can be extended to other applications as long as stable clusters with appropriate size and ligands can be obtained. Additionally, we need to note that the cluster-mediated process may differ from the process on an actual electrode surface. The difference between ligand-protected gold nanoclusters and a real electrocatalytic interface lies in whether the ligands affect the electron transfer process. Furthermore, when clusters detach from the electrode surface, their potential may change relative to that of the actual electrode, leading to differences from what actually occurs at the electrode interface. Therefore, our method should be regarded as a model system for monitoring interfacial intermediates, rather than a direct reproduction of the real electrode interface.

## Conclusion

In this work, we propose a DI-EC-MS strategy that converts inner-sphere electrocatalytic reactions, which are normally strictly confined to the electrode interface, into molecular entities that can be directly analyzed by mass spectrometry. By introducing an atomically precise [Au_9_(PPh_3_)_8_]^3+^ cluster as an MS-resolvable interfacial carrier, we construct a system in which electron transfer, interfacial adsorption and desorption, and cluster valence-state evolution are intrinsically coupled. In this way, interfacial reaction information can be preserved and read out in a stable form. This design effectively addresses the long-standing structural mismatch between where inner-sphere reactions occur and what mass spectrometry can detect. Based on this platform, we directly captured a hydride-type intermediate [Au_9_H(PPh_3_)_8_]^2+^, associated with proton-coupled electron transfer. We further investigated the potential-dependent reconstruction and growth of [Au_9_(PPh_3_)_8_]^3+^ and [Au_11_(PPh_3_)_8_]^3+^ in the presence of metal ions, providing molecular-level evidence for HER interfacial mechanisms. In the reduction of nitrosobenzene, we also mapped the full evolution pathway of the nitrosobenzene radical anion, from formation at the electrode interface to partial desorption into solution, revealing the dynamic connection between interface-confined chemistry and solution-phase processes. These results show that detachable interface mass spectrometry can not only capture short-lived inner-sphere intermediates but also resolve potential-dependent electrode interface reconstruction and distinguish interface-confined pathways from solution-phase routes, enabling *operando* molecular-level mechanistic analysis of electrochemical processes. This work redefines the electrode interface as a directly analyzable unit in electrochemical mass spectrometry, provides a new methodological framework for heterogeneous electrocatalysis studies, and lays the foundation for directly linking interfacial atomic structure with dynamic reaction pathways.

## Author contributions

Z. Wei, S. Chen and P. Li supervised the project, conceived the idea and designed the experiments. X. Zeng, P. Qiu, X. Cui, C. Zhu, C. Zeng and J. Chen carried out the experimental work. Z. Wei, S. Chen, P. Li, X. Zeng and P. Qiu analyzed the data and wrote the manuscript. All authors have given approval to the final version of the manuscript.

## Conflicts of interest

There are no conflicts to declare.

## Supplementary Material

SC-OLF-D6SC01821A-s001

## Data Availability

The supporting data has been provided as part of the supplementary information (SI). Supplementary information: experimental methods, supplementary figures and supplementary note. See DOI: https://doi.org/10.1039/d6sc01821a.
