# Supplementary material for: Direct visualization of inner-sphere electrocatalytic reactions as they occur at detachable electrochemical interfaces
Source: Chem Sci. 2026 Apr 27;17(25):12295–306. doi: 10.1039/d6sc01821a (PMC13187949; doi:10.1039/d6sc01821a)
Supplement: SC-017-D6SC01821A-s001 [file SC-017-D6SC01821A-s001.pdf]

Supplementary Information (SI) for Chemical Science.
This journal is © The Royal Society of Chemistry 2026

Supplementary Information

Direct Visualization of Inner-Sphere Electrocatalytic Reactions as They Occur at Detachable Electrochemical Interfaces

Xiangbiao Zeng, Peimeng Qiu, Xi Cui, Chengbiao Zhu, Chen Zeng, Jianxiong Chen, Peng Li,* Shengli Chen,* and Zhenwei Wei*

Hubei Key Lab. of Electrochemical Power Sources, College of Chemistry and Molecular Sciences, Wuhan University, Wuhan 430072 (China)

Contents

1. Experimental Section 5

1.1 Reagents and Materials 5

1.2 Fabrication of Electrospray ionization source emitters 5

1.3 In situ electrochemical mass spectrometry parameters 5

1.4 Other Instrument parameters 6

1.5 Synthesis Methods 7

1.6 DFT Calculations 8

2. Supplementary Note 8

3. Figures and Tables 10

Figure S1 Schematic diagram of the floating potential control module 10

Figure S2. Characterization data of Au NPs. (a) UV-vis absorption spectrum. (b) Positive ion mode mass spectrum. (c) Negative ion mode mass spectrum. (d) TEM image. (e) Particle size distribution histogram. 10

Figure S3. The structural evolution process of $[Au_9(PPh_3)_8]^{3+}$ at a distance of 9.5 Å from the Pt surface. 11

Figure S4. (a) Simulated and experimental isotope distribution patterns of $[Au_9(PPh_3)_8]^{2+}$. (b) Simulated and experimental isotope distribution patterns of $[Au_9(PPh_3)_8]^{+}$..12

Figure S5. Mass spectra of $[Au_9(PPh_3)_8](NO_3)_3$ after electrochemical reduction in (a) acetone, (b) dimethyl sulfoxide, and (c) *N*, *N*-dimethylformamide as the solvent. Among them, acetone has poor solubility, so a saturated solution of $[Au_9(PPh_3)_8](NO_3)_3$ was used for the experiment, while the concentration of $[Au_9(PPh_3)_8](NO_3)_3$ in other solvents was 500 ppm. 13

Figure S6. Mass spectra of $[Au_9(PPh_3)_8](NO_3)_3$ after electrochemical reduction in (a) methanol, (b) ethanol, and (c) acetonitrile-water (9:1, v/v) as the solvent. 14

Figure S7. DI-EC-MS was employed to monitor the electrochemical reaction of a mixed solution of $[Au_9(PPh_3)_8](NO_3)_3$ and DCOOD. (a) Online CV and corresponding MSV curves; (b) MS at 0 V *vs*. Fc/Fc$^+$; (c) MS at −2.0 V *vs*. Fc/Fc$^+$; (d) simulated and experimental isotope distribution patterns of $[Au_9D(PPh_3)_8]^{2+}$; (e) tandem MS of $[Au_9D(PPh_3)_8]^{2+}$. Experimental conditions: acetonitrile solution

containing $[Au_9(PPh_3)_8](NO_3)_3$ (500 ppm), DCOOD (1 mM), and Fc (0.1 mM). Scan rate: 20 mV s^{-1}. 15

Figure S8. (a) MSV of $[Au_9H(PPh_3)_8]^{2+}$ at different formic acid concentrations. (b) Relative intensity of $[Au_9H(PPh_3)_8]^{2+}$ at different formic acid concentrations (at −2.0 V *vs*. Fc/Fc^+). Experimental conditions: acetonitrile solution containing $[Au_9(PPh_3)_8](NO_3)_3$ (500 ppm), HCOOH (0–100 mM), and Fc (0.1 mM). Scan rate: 20 mV s^{-1}. 16

Figure S9. (a) MSV of $[Au_9(PPh_3)_8]^{2+}$ at different formic acid concentrations. (b) MSV of $[Au_9(PPh_3)_8]^{+}$ at different formic acid concentrations. Experimental conditions: acetonitrile solution containing $[Au_9(PPh_3)_8](NO_3)_3$ (500 ppm), HCOOH (0–100 mM), and Fc (0.1 mM). Scan rate: 20 mV s^{-1}. 17

Figure S10. (a) MS at 0 V (*vs*. Fc/Fc^+). (b) MS at −1.15 V (*vs*. Fc/Fc^+) with HCOOH (1 mM). (c) Simulated and experimental isotope distribution patterns of $[Au_{10}(PPh_3)_8]^{2+}$. (d) Proposed pathway for the formation of $[Au_{10}(PPh_3)_8]^{2+}$ and MSV of $[Au_{10}(PPh_3)_8]^{2+}$ under the condition of 1 mM HCOOH. (e) MS at −2.0 V (*vs*. Fc/Fc^+) with HCOOH (10 mM). (f) MSV of $[Au_{10}(PPh_3)_8]^{2+}$ under the condition of 10 mM HCOOH. 18

Figure S11. (a) MS of $[Au_9(PPh_3)_8](NO_3)_3$. (b) MS after the addition of 10 mM HCOOH and 0.5mM $Au(PPh_3)NO_3$. (c) Simulated and experimental isotope distribution patterns of $[Au_{11}(PPh_3)_8]^{3+}$. (d) Relative intensity of $[Au_{11}(PPh_3)_8]^{3+}$ at different formic acid concentrations. 18

Figure S12. Simulated and experimental isotope distribution patterns of (a) $[HAu_{12}(PPh_3)_9]^{3+}$; (b) $[H_2Au_{13}(PPh_3)_{10}]^{3+}$; (c) $[H_3Au_{14}(PPh_3)_{10}]^{3+}$; (d) $[CH_3CN+Au(PPh_3)]^{+}$. Among them, the distortion of the $[HAu_{12}(PPh_3)_9]^{3+}$ isotopic distribution pattern is due to interference from the isotopic peaks of $[Au_8(PPh_3)_6]^{2+}$ (m/z 1574.6400)..19

Figure S13. EC reaction of a mixed solution containing $[Au_9(PPh_3)_8](NO_3)_3$, DCOOD, and $Au(PPh_3)NO_3$ was monitored using DI-EC-MS. (a) MS at 0 V *vs*. Fc/Fc^+. (b) MS at −1.7 V *vs*. Fc/Fc^+. (c) Simulated and experimental isotope distribution patterns of $[DAu_{12}(PPh_3)_9]^{3+}$. (d) Simulated and experimental isotope distribution patterns of $[D_2Au_{13}(PPh_3)_{10}]^{3+}$. (e) Simulated and experimental isotope distribution patterns of $[D_3Au_{14}(PPh_3)_{10}]^{3+}$. (f) CV. (g) MSV of selected ions. (h) Proposed mechanism for the growth from $[Au_{11}(PPh_3)_8]^{3+}$ to

$[D_3Au_{14}(PPh_3)_{10}]^{3+}$. Experimental conditions: Acetonitrile solution containing $[Au_9(PPh_3)_8](NO_3)_3$ (500 ppm), DCOOD (10 mM), $Au(PPh_3)NO_3$ (0.5 mM) and Fc (0.1 mM). Scan rate: 20 mV s^{-1}. 20

Figure S14. EC reaction of a mixed solution containing $[Au_9(PPh_3)_8](NO_3)_3$ and and PhNO was monitored using FE-ESI-MS in negative ion mode. (a) MS at −2.0 V *vs*. Fc/Fc^+. (b) MSV of 107.0377. 21

Figure S15. Positive ion mode MS of a mixed solution containing $[Au_9(PPh_3)_8](NO_3)_3$ and PhNO (a) at 0 V *vs*. Fc/Fc^+ and (b) at −1.7 V *vs*. Fc/Fc^+. No product signal was detected in either spectrum...........................21

Figure S16. EC reaction of a mixed solution containing $[Au_9(PPh_3)_8](NO_3)_3$, PhNO, and HCOOH was monitored using FE-ESI-MS. (a) Proposed mechanism for the hydrogenation reduction of PhNO; (b) online CV and corresponding MSV curves; (c) MS at 0 V *vs*. Fc/Fc^+; (d) MS at −1.70 V *vs*. Fc/Fc^+. Experimental conditions: acetonitrile solution containing $[Au_9(PPh_3)_8](NO_3)_3$ (500 ppm), HCOOH (1 mM), and Fc (0.1 mM). Scan rate: 20 mV s^{-1}. 22

Figure S17. MS of the mixed solution containing $[Au_9(PPh_3)_8](NO_3)_3$, PhNO, and HCOOH at −2.0 V *vs*. Fc/Fc^+. The spectrum shows that $[Au_9(PPh_3)_7+PhNO]^{2+}$ was not detected. 23

Reference...24

1. Experimental Section

1.1 Reagents and Materials

Sodium borohydride ($NaBH_4$) was purchased from Chengdu Kelong Chemical Reagent Factory. Chloro(triphenylphosphine)gold(I) ($AuPPh_3Cl$), sodium citrate, ferrocene (Fc), and nitrosobenzene (PhNO) were purchased from Shanghai Bide Pharmatech Co., Ltd. Silver nitrate ($AgNO_3$) and formic acid-d$_2$ (DCOOD) were purchased from Shanghai Aladdin Biochemical Technology Co., Ltd. Methanol (CH_3OH) and acetonitrile (CH_3CN, HPLC grade) were purchased from Merck. Hydrogen tetrachloroaurate(III) trihydrate ($HAuCl_4 \cdot 3H_2O$) and formic acid (HCOOH) were purchased from Shanghai Macklin Biochemical Co., Ltd. Deuterated chloroform (CD_3Cl) was purchased from Sigma-Aldrich(Shanghai)Trading Co.,Ltd. Acetone (CH_3COCH_3), dimethyl sulfoxide (DMSO), and *N*, *N*-dimethylformamide (DMF) were purchased from Sinopharm Chemical Reagent Co., Ltd. Ethanol (CH_3CH_2OH) was purchased from FTSCI Science and Technology Co., Ltd. Ultrapure water was prepared using a Milli-Q ultrapure water system.

Platinum wire (diameter: 0.1 mm) and silver wire (diameter: 0.15 mm) were purchased from Sinopharm Chemical Reagent Co., Ltd.

1.2 Fabrication of Electrospray ionization source emitters

Nano-electrospray ionization source (nESI) emitters were pulled from borosilicate glass tube by using a P-97 microelectrode puller (Sutter Instrument, Co., Ltd., USA). In all experiments, the nESI emitters used had a tip diameter of approximately 3 μm. The pulling parameters for these emitters were as follows: HEAT = 610, PULL = 10, VEL = 9, DEL = 225, P = 200.

1.3 In situ electrochemical mass spectrometry parameters

We have improved the previously developed spatiotemporally coupled floating in situ electrochemical mass spectrometry setup by introducing a floating potential control module, enabling the device to vary the electrochemical reaction potential (from −5V to 5V) under kilovolt-level floating high voltage[1]. This floating potential module was provided by Suzhou Fortune Precision Instrument Co., Ltd. The floating potential control module is powered by an independent portable power supply and is based on a

“floating ground” architecture. In this design, the reference ground (GND) terminal of the power supply is connected to the high voltage required for electrospray ionization in mass spectrometry, ensuring stable operation of the entire electrochemical control system under high common-mode voltage conditions. The module is equipped with built-in high-voltage protection circuits (e.g., transient voltage suppression diodes), which effectively prevent damage to internal components caused by high-voltage breakdown. A schematic illustration of the module is shown in Figure S1. Consequently, with the incorporation of the floating potential module, the cyclic voltammetry curve scanning and mass spectrometry monitoring can be carried out synchronously. In the electrochemical reaction, two platinum wires were used as the working and counter electrodes, respectively, and a silver wire served as the reference electrode. The potential was calibrated using the Fc/Fc^{+} redox couple. The platinum wires were insulated with polyimide capillaries to prevent short circuits. The electrodes were inserted into the front end of the nESI emitter, as shown in Figure 1a.

Mass spectrometry data were acquired using an Orbitrap Ascend Tribrid mass spectrometer (Thermo Fisher Scientific Co., Ltd., USA). The ion transmission tube temperature was set to 275 °C, and the energy of the S-lens was set to 60%. The number of microscans was set to 1, with a mass resolution of 60,000. For high-resolution tandem MS experiments, the isolation window was set to 1.0 Da, and normalized collision energies from 22 to 25 (units were defined by the instrument manufacturer, the collision gas was helium). All MS data were analyzed using Qual Browser (Xcalibur workstation, Version 4.2.47).

1.4 Other Instrument parameters

UV-Vis spectra were acquired using a Hitachi UH5700 UV-Vis-NIR spectrophotometer. For gold nanoparticles, water was used as the solvent, while ethanol was used as the solvent for gold nanoclusters. ^{31}P NMR spectra were recorded on a Bruker AVANCE III HD (400 MHz) spectrometer using CD_3Cl as the solvent. Transmission electron microscopy (TEM) characterization was performed on a JEOL JEM-2100 instrument.

1.5 Synthesis Methods

1.5.1 Synthesis of Gold Nanoparticles

Gold nanoparticles (AuNPs) were synthesized according to a previously reported method [2]. In a typical procedure, 50 mL of an aqueous $HAuCl_4$ solution (1 mM) was heated to boiling under vigorous stirring. Subsequently, 5 mL of a sodium citrate solution (38.8 mM) was rapidly introduced into the boiling mixture. The reaction was maintained under boiling conditions for 10 minutes, after which heating was ceased while stirring continued for another 15 minutes. Once the solution had cooled to room temperature, it was filtered through a 0.22 μm syringe filter and stored for further use.

1.5.2 Synthesis of $Au(PPh_3)NO_3$

$Au(PPh_3)NO_3$ was synthesized following a previously reported method[3]. To a 65 mL methanol solution containing 4.04 mmol of silver nitrate, a 20 mL dichloromethane solution containing 2.02 mmol of $Au(PPh_3)Cl$ was added dropwise with stirring. The reaction mixture was stirred at room temperature for 1 hour. After centrifugation to remove the precipitate, the supernatant was concentrated to dryness under vacuum. The resulting solid was redissolved in 30 mL of dichloromethane, and the mixture was centrifuged again to remove precipitate. The clear supernatant was then evaporated to dryness under vacuum. The obtained solid was dissolved in a minimal amount of dichloromethane, and three volumes of anhydrous ethanol were added. The mixture was stirred slowly under a nitrogen atmosphere for 1 hour, yielding white crystals. The crystals were collected, washed several times with absolute ethanol and diethyl ether, and finally dried.

1.5.3 Synthesis of $[Au_9(PPh_3)_8](NO_3)_3$

$[Au_9(PPh_3)_8](NO_3)_3$ was synthesized according to a previously reported method [4]. A solution of $NaBH_4$ (0.24 mmol) in ethanol (12 mL) was added dropwise to a stirred solution of $Au(PPh_3)NO_3$ (0.95 mmol) in ethanol (20 mL). The mixture was stirred at room temperature for 2 hours and then filtered. The filtrate was evaporated to dryness under vacuum. The resulting solid was dissolved in dichloromethane (2.5 mL) and filtered. The filtrate was again evaporated to dryness under vacuum. The product was washed several times with tetrahydrofuran and hexane to obtain a dark green powder.

1.6 DFT Calculations

Spin-polarized density function theory (DFT) calculations were performed using the Vienna Ab Initio Simulation Package (VASP) [5] with the generalized gradient approximation (GGA) of Perdew-Burke-Ernzerhof (PBE) [6]. A plane-wave cutoff energy of 450 eV was used. The convergence threshold was 10^{-5} eV in energy and 0.02 eV $Å^{-1}$ in force for electronic relaxation and ion relaxation, respectively. Long-range dispersion interactions were described by the DFT-D3 method of Grimme with zero damping [7]. The Brillouin zone was sampled at the Γ point using a 1 × 1 × 1 Monkhorst-Pack grid. The Pt(111) surface was modeled by a two-layer slab with supercell size of p(4 × 7), in which a = 19.22 Å and b = 19.42 Å. The thickness of the vacuum layer is ~1.5 nm. To reduce the computational cost, the experimentally used $[Au_9(PPh_3)_8]^{3+}$ cluster was modeled as $[Au_9(PPh_3)_4(PH_3)_4]^{3+}$, in which four PPh_3 ligands were replaced by PH_3 while retaining four intact PPh_3 ligands. To properly describe the interfacial electric double layer, the intact PPh_3 ligands were oriented toward the Pt electrode surface. The adsorption configuration of the $[Au_9(PPh_3)_4(PH_3)_4]^{3+}$ cluster on Pt was determined by placing the cluster at several different vertical distances of 9.5, 11.0, 12.0, and 13.0 Å, defined as the distance between the surface Pt atoms and the central Au atom of the cluster, and performing structural optimizations for each initial position. The lowest energy structure obtained in this way was taken as the most stable adsorption configuration and was used for subsequent calculations under both neutral and negatively charged surface conditions. The negative surface charge was introduced by adjusting the total number of electrons in the slab model to yield a net surface charge density of −12.85 μC cm^{-2}, which was chosen to qualitatively mimic a negatively charged Pt surface under −0.606 V versus the standard hydrogen electrode (−0.606 V was derived from the experimental value of −1.23 V *vs*. Fc/Fc^+ based on a previously reported conversion method[8]).

2. Supplementary Note

The density of the gold nanoparticles was approximated using the known density of solid gold, 19.32 g/cm^3.

Given:

Nanoparticle diameter d = 15.8 nm = 1.58×10^{-8} m

Radius $r = d/2 = 7.9 \times 10^{-9}$ m = 7.9×10^{-7} cm

Volume of a spherical nanoparticle $V = (4/3)\pi r^3 = 2.06 \times 10^{-18}$ cm³

Mass of a single nanoparticle $m = \rho V = 3.99 \times 10^{-17}$ g

Converting mass to daltons (Da), where 1 Da = 1.66×10^{-24} g

$m = (3.99 \times 10^{-17}$ g$) / (1.66 \times 10^{-24}$ g/Da$) = 2.40 \times 10^{7}$ Da

The calculated mass indicates that the gold nanoparticles are relatively large, which exceeds the typical mass range detectable by conventional mass spectrometry. Therefore, Au NPs are difficult to detect via mass spectrometry under standard experimental conditions.

3. Figures and Tables

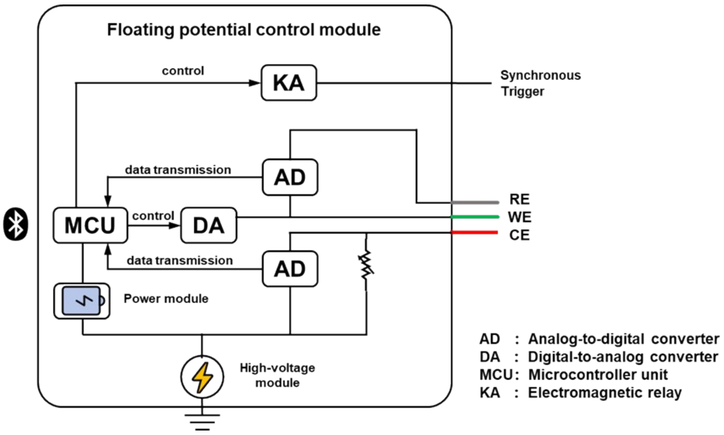

Figure S1 Schematic diagram of the floating potential control module

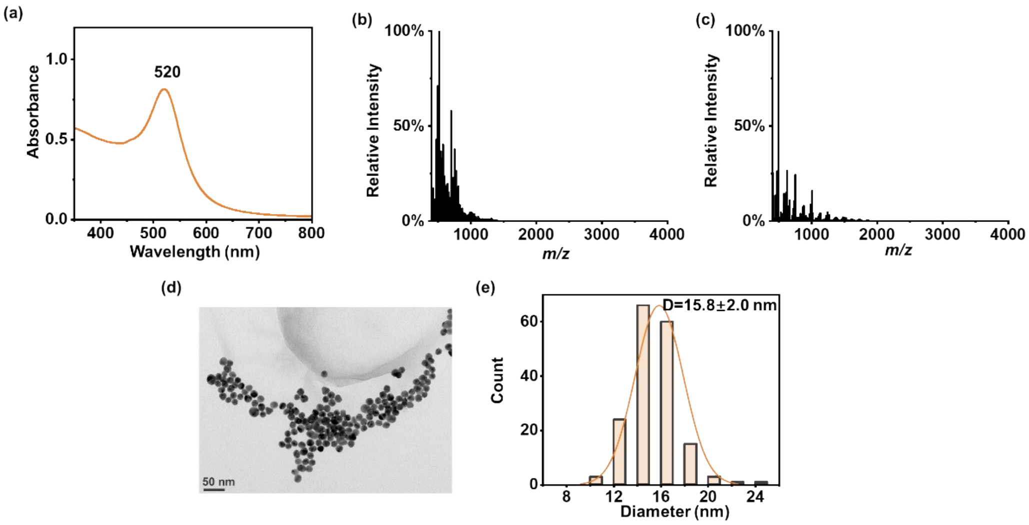

Figure S2. Characterization data of Au NPs. (a) UV-vis absorption spectrum. (b) Positive ion mode mass spectrum. (c) Negative ion mode mass spectrum. (d) TEM image. (e) Particle size distribution histogram.

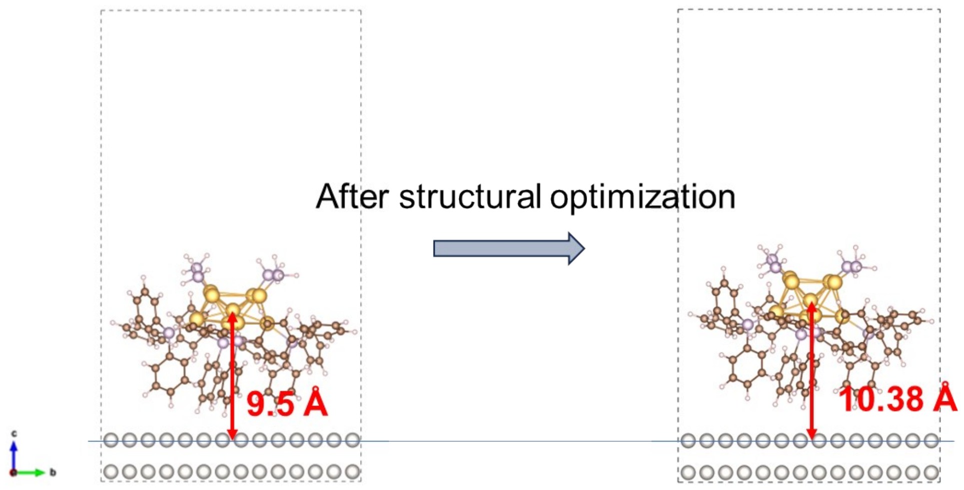

Figure S3. The structural evolution process of $[Au_9(PPh_3)_8]^{3+}$ at a distance of 9.5 Å from the Pt surface.

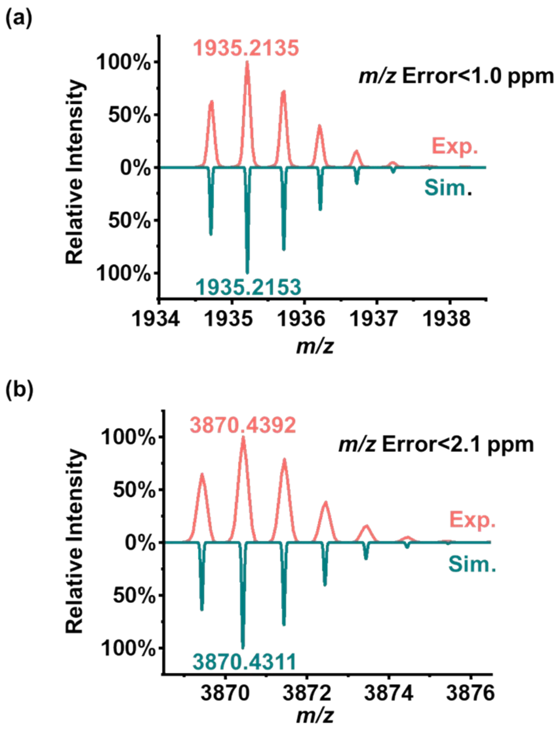

Figure S4. (a) Simulated and experimental isotope distribution patterns of $[Au_9(PPh_3)_8]^{2+}$. (b) Simulated and experimental isotope distribution patterns of $[Au_9(PPh_3)_8]^{+}$.

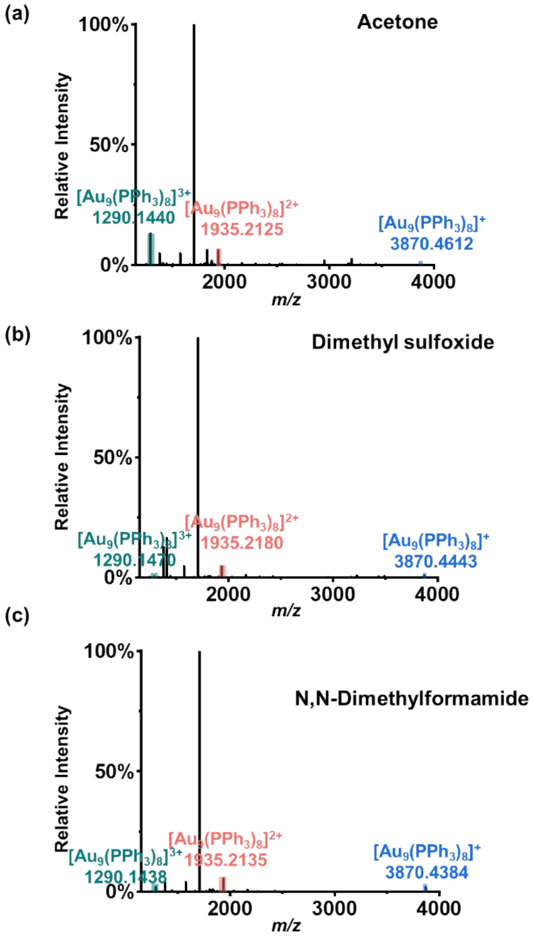

Figure S5. Mass spectra of $[Au_9(PPh_3)_8](NO_3)_3$ after electrochemical reduction in (a) acetone, (b) dimethyl sulfoxide, and (c) *N*, *N*-dimethylformamide as the solvent. Among them, acetone has poor solubility, so a saturated solution of $[Au_9(PPh_3)_8](NO_3)_3$ was used for the experiment, while the concentration of $[Au_9(PPh_3)_8](NO_3)_3$ in other solvents was 500 ppm.

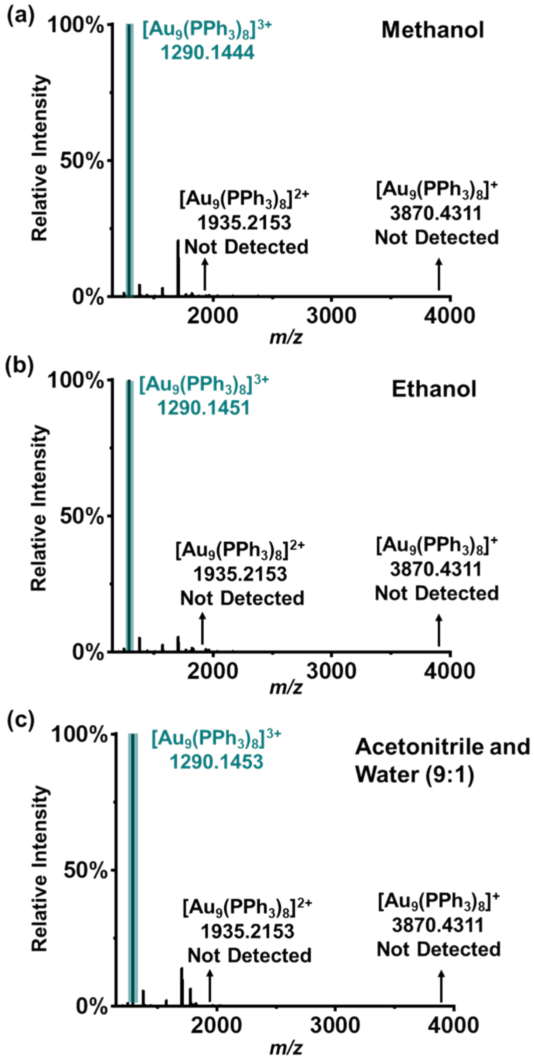

Figure S6. Mass spectra of $[Au_9(PPh_3)_8](NO_3)_3$ after electrochemical reduction in (a) methanol, (b) ethanol, and (c) acetonitrile-water (9:1, v/v) as the solvent.

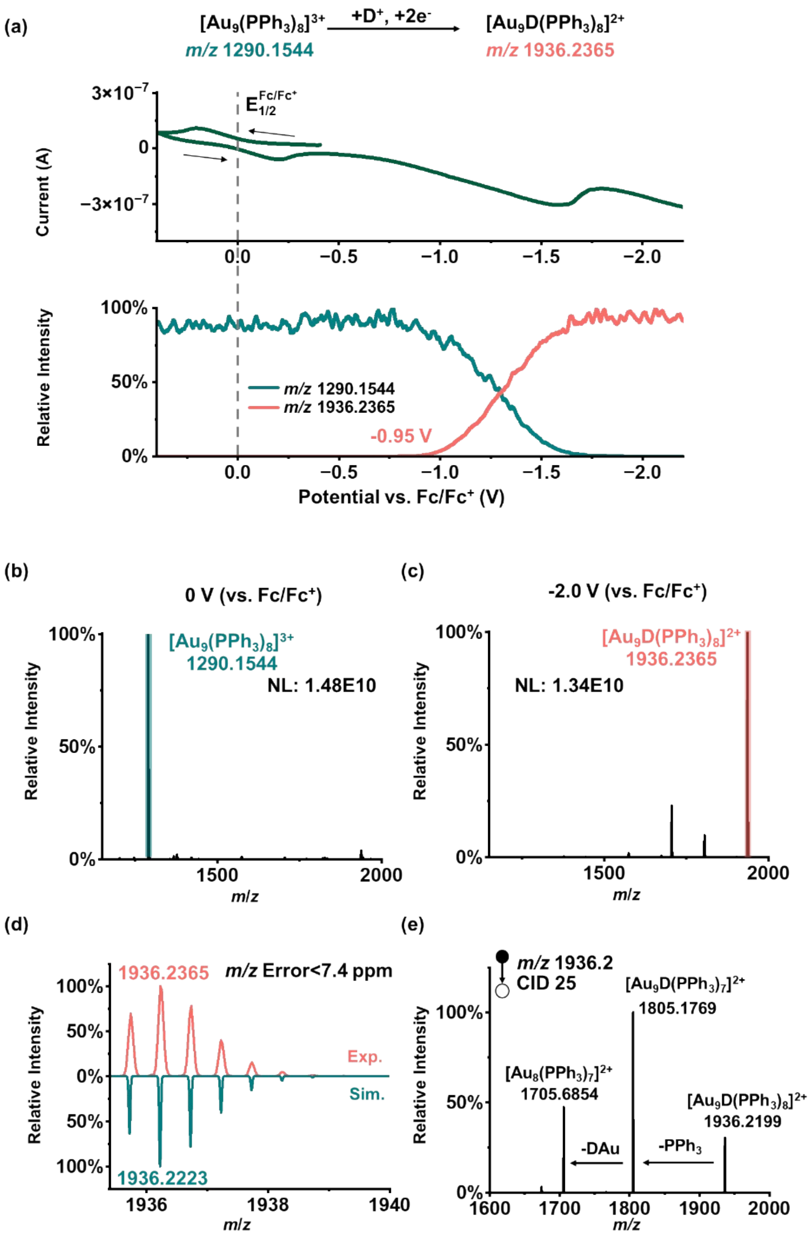

Figure S7. DI-EC-MS was employed to monitor the electrochemical reaction of a mixed solution of $[Au_9(PPh_3)_8](NO_3)_3$ and DCOOD. (a) Online CV and corresponding MSV curves; (b) MS at 0 V *vs*. Fc/Fc^+; (c) MS at −2.0 V *vs*. Fc/Fc^+; (d) simulated and experimental isotope distribution patterns of $[Au_9D(PPh_3)_8]^{2+}$; (e) tandem MS of $[Au_9D(PPh_3)_8]^{2+}$. Experimental conditions: acetonitrile solution containing $[Au_9(PPh_3)_8](NO_3)_3$ (500 ppm), DCOOD (1 mM), and Fc (0.1 mM). Scan rate: 20 mV s^{-1}.

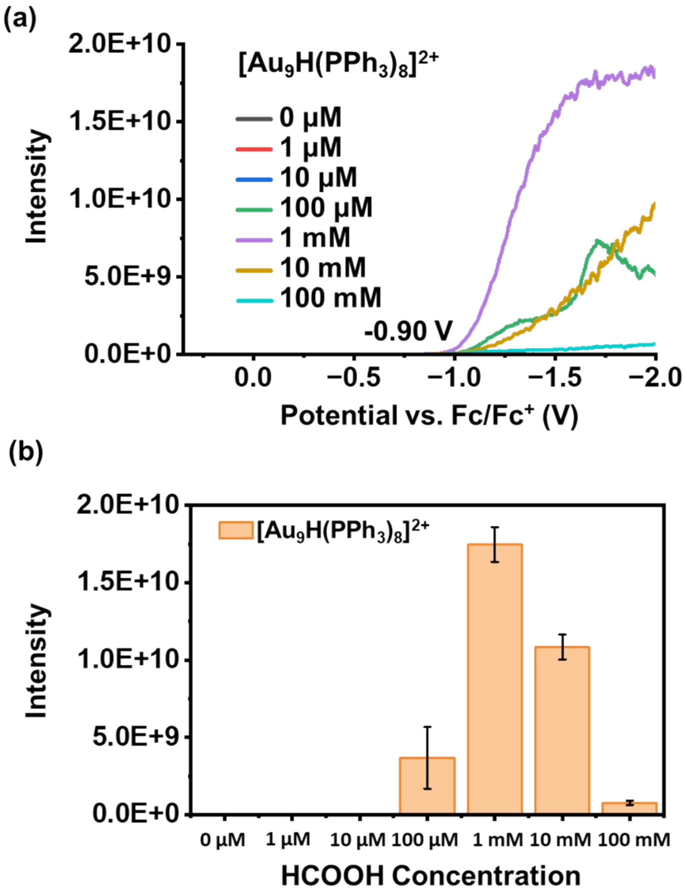

Figure S8. (a) MSV of $[Au_9H(PPh_3)_8]^{2+}$ at different formic acid concentrations. (b) Relative intensity of $[Au_9H(PPh_3)_8]^{2+}$ at different formic acid concentrations (at −2.0 V *vs*. Fc/Fc^+). Experimental conditions: acetonitrile solution containing $[Au_9(PPh_3)_8](NO_3)_3$ (500 ppm), HCOOH (0–100 mM), and Fc (0.1 mM). Scan rate: 20 mV s^{-1}.

(a)

(b)

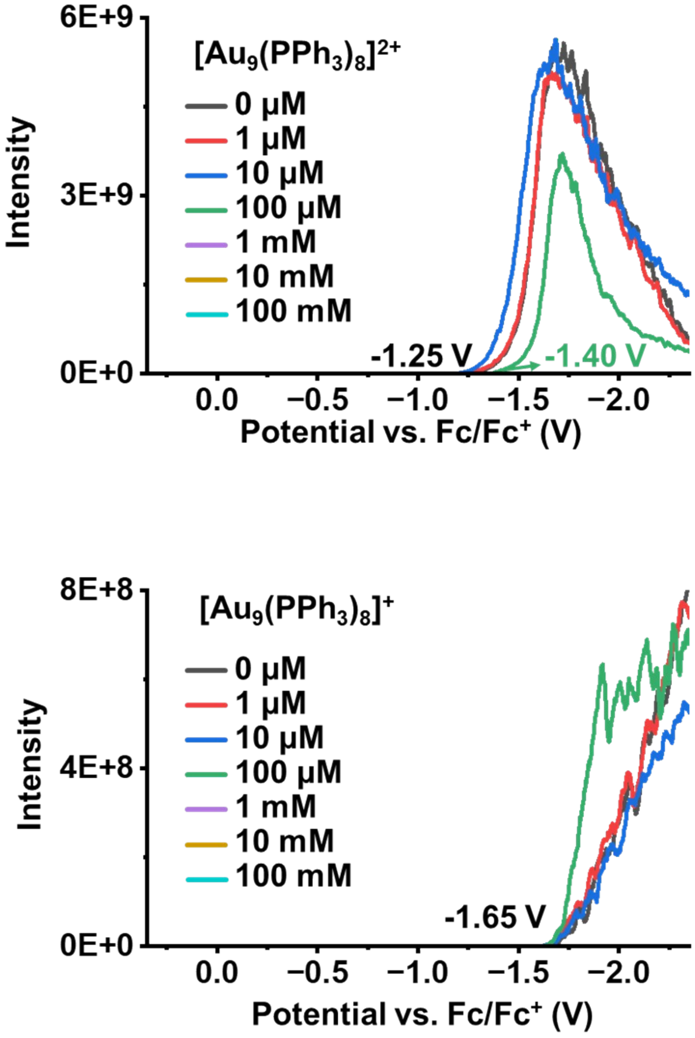

Figure S9. (a) MSV of $[Au_9(PPh_3)_8]^{2+}$ at different formic acid concentrations. (b) MSV of $[Au_9(PPh_3)_8]^{+}$ at different formic acid concentrations. Experimental conditions: acetonitrile solution containing $[Au_9(PPh_3)_8](NO_3)_3$ (500 ppm), HCOOH (0–100 mM), and Fc (0.1 mM). Scan rate: 20 mV s^{-1}.

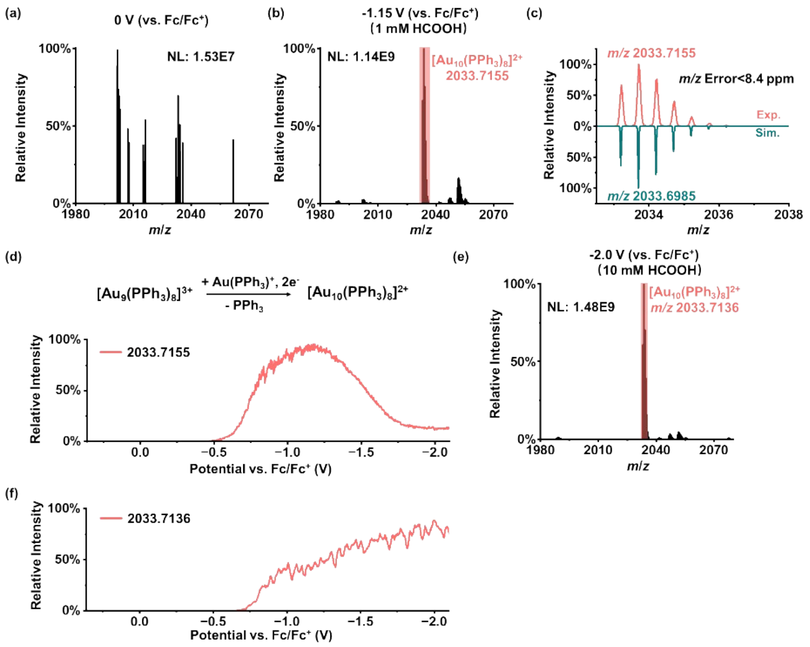

Figure S10. (a) MS at 0 V (*vs*. Fc/Fc$^+$). (b) MS at −1.15 V (*vs*. Fc/Fc$^+$) with HCOOH (1 mM). (c) Simulated and experimental isotope distribution patterns of $[Au_{10}(PPh_3)_8]^{2+}$. (d) Proposed pathway for the formation of $[Au_{10}(PPh_3)_8]^{2+}$ and MSV of $[Au_{10}(PPh_3)_8]^{2+}$ under the condition of 1 mM HCOOH. (e) MS at −2.0 V (*vs*. Fc/Fc$^+$) with HCOOH (10 mM). (f) MSV of $[Au_{10}(PPh_3)_8]^{2+}$ under the condition of 10 mM HCOOH.

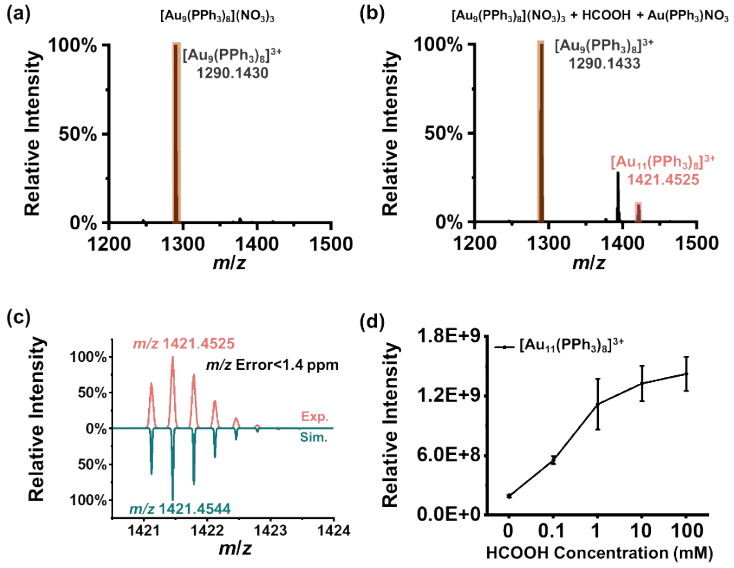

Figure S11. (a) MS of $[Au_9(PPh_3)_8](NO_3)_3$. (b) MS after the addition of 10 mM HCOOH and 0.5mM $Au(PPh_3)NO_3$. (c) Simulated and experimental isotope distribution patterns of $[Au_{11}(PPh_3)_8]^{3+}$. (d) Relative intensity of $[Au_{11}(PPh_3)_8]^{3+}$ at different formic acid concentrations.

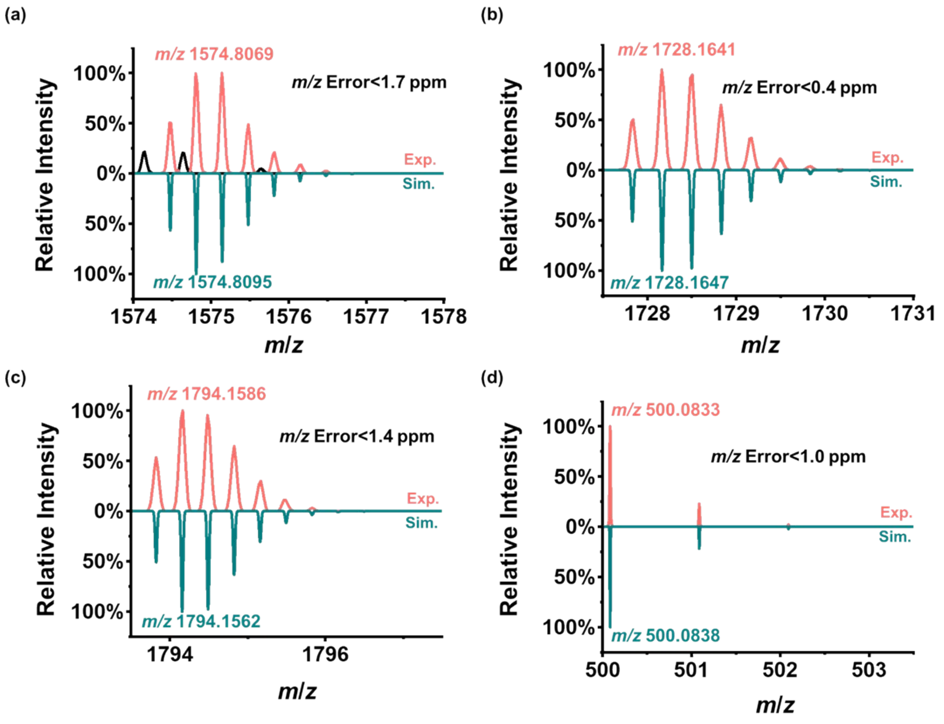

Figure S12. Simulated and experimental isotope distribution patterns of (a) $[HAu_{12}(PPh_3)_9]^{3+}$; (b) $[H_2Au_{13}(PPh_3)_{10}]^{3+}$; (c) $[H_3Au_{14}(PPh_3)_{10}]^{3+}$; (d) $[CH_3CN{+}Au(PPh_3)]^{+}$. Among them, the distortion of the $[HAu_{12}(PPh_3)_9]^{3+}$ isotopic distribution pattern is due to interference from the isotopic peaks of $[Au_8(PPh_3)_6]^{2+}$ (m/z 1574.6400).

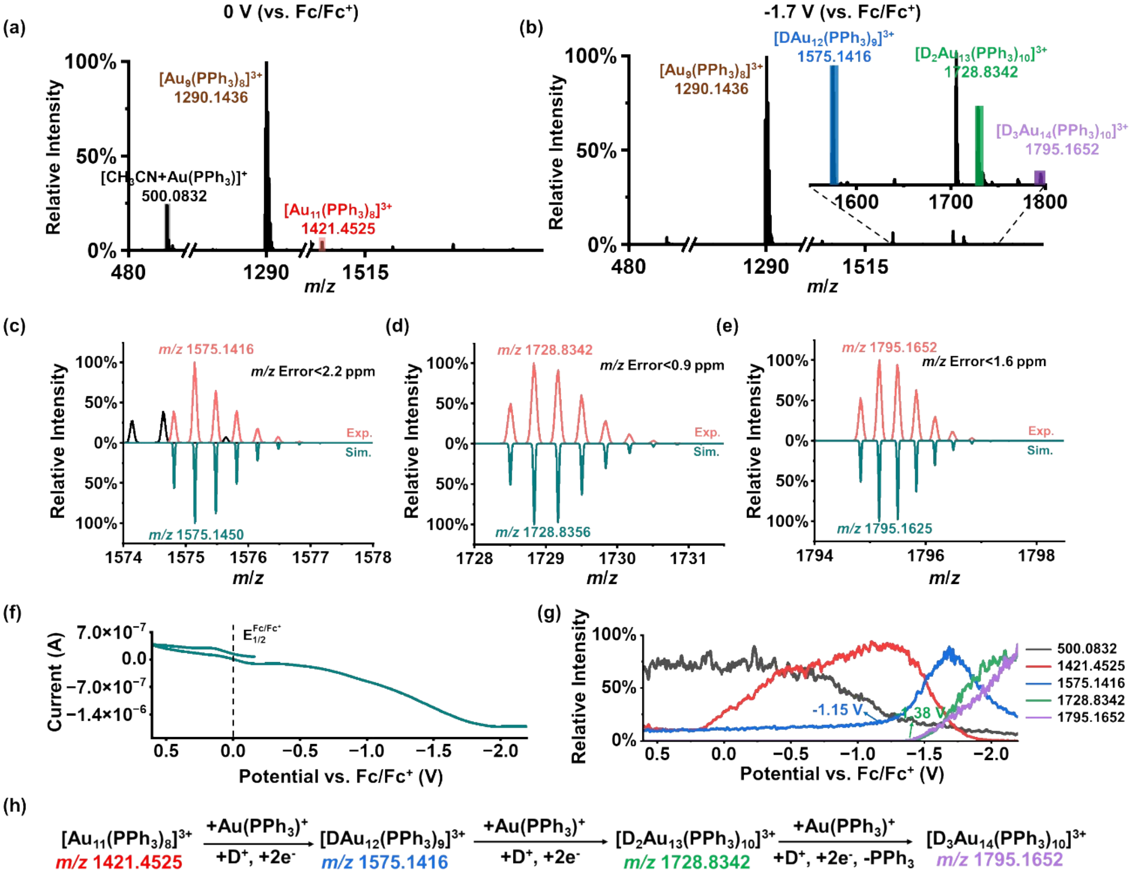

Figure S13. EC reaction of a mixed solution containing $[Au_9(PPh_3)_8](NO_3)_3$, DCOOD, and $Au(PPh_3)NO_3$ was monitored using DI-EC-MS. (a) MS at 0 V *vs*. Fc/Fc^+. (b) MS at −1.7 V *vs*. Fc/Fc^+. (c) Simulated and experimental isotope distribution patterns of $[DAu_{12}(PPh_3)_9]^{3+}$. (d) Simulated and experimental isotope distribution patterns of $[D_2Au_{13}(PPh_3)_{10}]^{3+}$. (e) Simulated and experimental isotope distribution patterns of $[D_3Au_{14}(PPh_3)_{10}]^{3+}$. (f) CV. (g) MSV of selected ions. (h) Proposed mechanism for the growth from $[Au_{11}(PPh_3)_8]^{3+}$ to $[D_3Au_{14}(PPh_3)_{10}]^{3+}$. Experimental conditions: Acetonitrile solution containing $[Au_9(PPh_3)_8](NO_3)_3$ (500 ppm), DCOOD (10 mM), $Au(PPh_3)NO_3$ (0.5 mM) and Fc (0.1 mM). Scan rate: 20 mV s^{-1}.

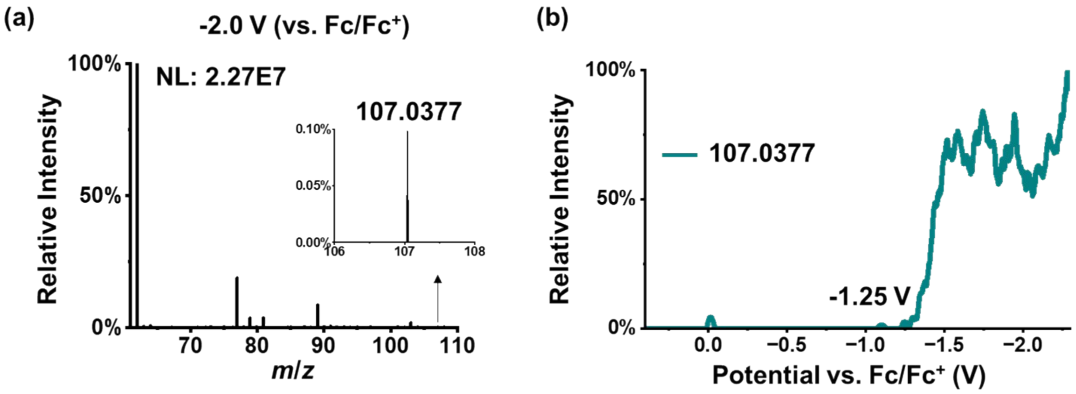

Figure S14. EC reaction of a mixed solution containing $[Au_9(PPh_3)_8](NO_3)_3$ and and PhNO was monitored using FE-ESI-MS in negative ion mode. (a) MS at −2.0 V *vs*. Fc/Fc^+. (b) MSV of 107.0377.

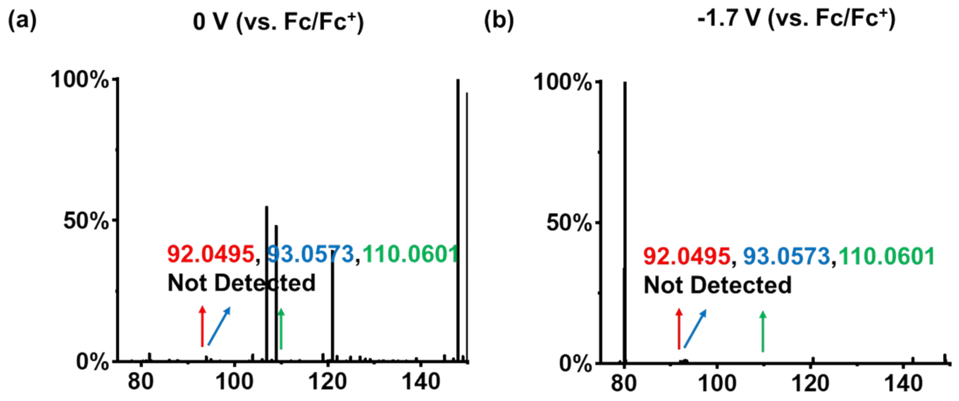

Figure S15. Positive ion mode MS of a mixed solution containing $[Au_9(PPh_3)_8](NO_3)_3$ and PhNO (a) at 0 V *vs*. Fc/Fc^+ and (b) at −1.7 V *vs*. Fc/Fc^+. No product signal was detected in either spectrum.

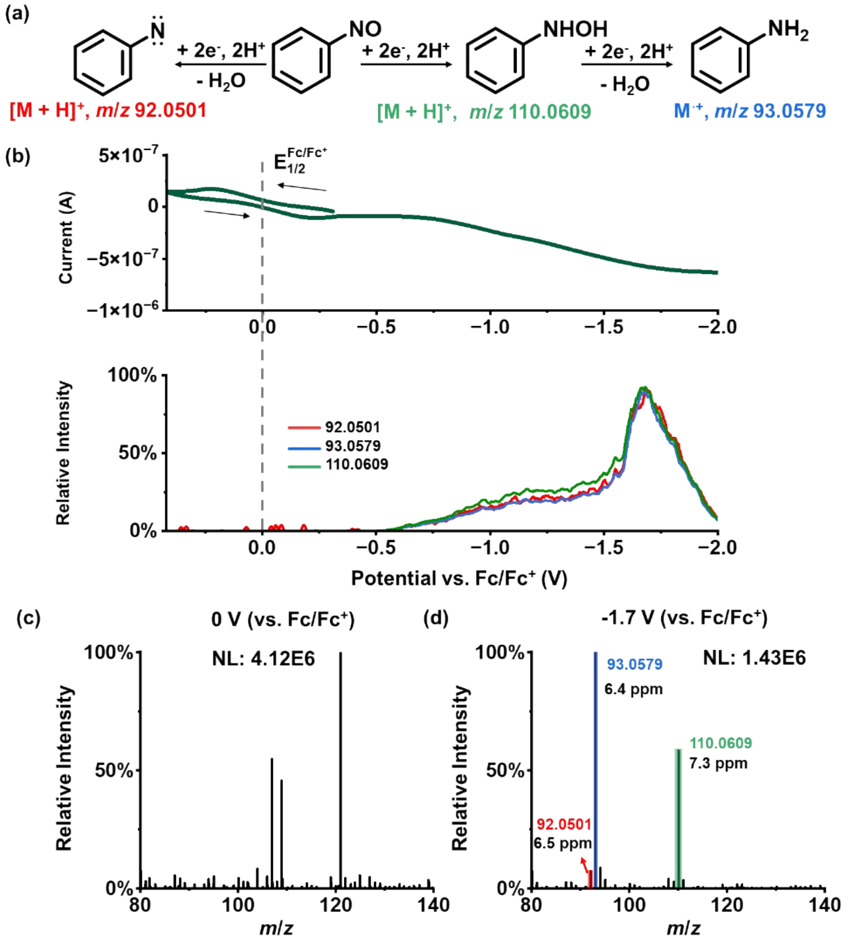

Figure S16. EC reaction of a mixed solution containing $[Au_9(PPh_3)_8](NO_3)_3$, PhNO, and HCOOH was monitored using FE-ESI-MS. (a) Proposed mechanism for the hydrogenation reduction of PhNO; (b) online CV and corresponding MSV curves; (c) MS at 0 V *vs*. Fc/Fc^+; (d) MS at −1.70 V *vs*. Fc/Fc^+. Experimental conditions: acetonitrile solution containing $[Au_9(PPh_3)_8](NO_3)_3$ (500 ppm), HCOOH (1 mM), and Fc (0.1 mM). Scan rate: 20 mV s^{-1}.

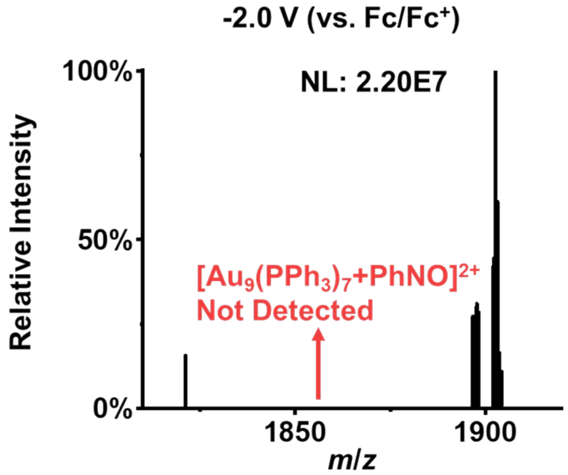

Figure S17. MS of the mixed solution containing $[Au_9(PPh_3)_8](NO_3)_3$, PhNO, and HCOOH at −2.0 V *vs*. Fc/Fc^+. The spectrum shows that $[Au_9(PPh_3)_7+PhNO]^{2+}$ was not detected.

Reference

1. J. Chen, X. Wang, X. Cui, Y. Li, Y. Feng and Z. Wei, In Situ Probing and Identification of Electrochemical Reaction Intermediates by Floating Electrolytic Electrospray Mass Spectrometry, *Angew. Chem. Int. Ed.*, 2023, **62**, e202219302.
2. K. C. Crabar, R. G. Freeman, M. B. Hommer and M. J. Natan, Preparation and Characterization of Au Colloid Monolayers, *Anal. Chem.*, 1995, **67**, 735-743.
3. A. M. Mueting, B. D. Alexander, P. D. Boyle, A. L. Casalnuovo, L. N. Ito, B. J. Johnson, L. H. Pignolet, M. Leeaphon, K. E. Meyer, R. A. Walton, D. M. Heinekey and T. G. P. Harper, Mixed-Metal-Gold Phosphine Cluster Compounds, *Inorg. Synth.*, 1992, **29**, 279-298.
4. F. Wen, U. Englert, B. Gutrath and U. Simon, Crystal Structure, Electrochemical and Optical Properties of $[Au_9(PPh_3)_8](NO_3)_3$, *Eur. J. Inorg. Chem.*, 2007, **2008**, 106-111.
5. G. Kresse and J. Furthmüller, Efficiency of ab-initio total energy calculations for metals and semiconductors using a plane-wave basis set, *Comput. Mater. Sci.*, 1996, **6**, 15-50.
6. J. P. Perdew, K. Burke and M. Ernzerhof, Generalized Gradient Approximation Made Simple [Phys. Rev. Lett. 77, 3865 (1996)], *Phys. Rev. Lett.*, 1997, **78**, 1396.
7. S. Grimme, J. Antony, S. Ehrlich and H. Krieg, A consistent and accurateab initioparametrization of density functional dispersion correction (DFT-D) for the 94 elements H-Pu, *J. Chem. Phys.*, 2010, **132**, 154104.
8. V. V. Pavlishchuk and A. W. Addison, Conversion constants for redox potentials measured versus different reference electrodes in acetonitrile solutions at 25°C, *Inorg. Chim. Acta*, 2000, **298**, 97-102.
